# Socioeconomic inequalities and the COVID-19 pandemic in France: Territorial analyzes based on epidemic wave and metropolitan area

**DOI:** 10.1371/journal.pone.0348201

**Published:** 2026-05-08

**Authors:** Luka Canton, Pierre Schalkwijk, Jordi Landier, Stanislas Rebaudet, Emilie Mosnier, Pascal Handschumacher, Stève Nauleau, Philippe Malfait, Ludivine Launay, Cyrille Delpierre, Michelle Kelly-Irving, Sabira Smaili, Stephanie Vandentorren, Jean Gaudart

**Affiliations:** 1 Aix Marseille Univ, Inserm, IRD, SESSTIM, Sciences Economiques and Sociales de la Santé and Traitement de l’Information Médicale, ISSPAM, Marseille, France; 2 Hôpital Européen, Marseille, France; 3 Service de Maladies Infectieuses et Tropicales, Sites Sud, CHU de La Réunion, La Réunion, France; 4 Agence Régionale de la Santé Provence Alpes Côte d’Azur, Marseille, France; 5 Santé Publique France Cellule Régionale Paca-Corse, Marseille, France; 6 U1086 Inserm Anticipe, Avenue Général Harris, Caen Cedex, France; 7 University Hospital of Caen, Caen Cedex, France; 8 Plateforme MapInMed, US PLATON, Avenue Général Harris, Caen Cedex, France; 9 Centre d’Epidémiologie et de Recherche en santé des POPulations (CERPOP) UMR1295, Equity team, Inserm, Université Toulouse III Paul Sabatier, Toulouse, France; 10 Santé Publique France, Saint-Maurice, France; 11 University of Bordeaux, INSERM UMR 1219–Bordeaux Population Health, Bordeaux, France; The University of Queensland, AUSTRALIA

## Abstract

**Background:**

Previous studies have highlighted the relationship between socioeconomic inequalities and the general population’s risk of contracting or dying from COVID-19 during the 2020−2023 pandemic. In France, socioeconomic inequalities vary across metropolitan areas; few studies have investigated whether this variation explains the spatial disparities observed in COVID-19 incidence and testing rates during the pandemic. We examined the relationship between socioeconomic profiles and these two rates across all 22 metropolitan areas in France for eight of the country’s nine epidemic waves.

**Methods:**

For each metropolitan area, we used socioeconomic variables from census data to define socioeconomic profiles through principal component clustering. We then used spatialized generalised additive mixed models to analyze associations between these profiles and both testing and incidence rates, for each epidemic wave from July 2020 to March 2023. Finally, we performed meta-regressions to study the distribution of testing and incidence rate ratios among the various socioeconomically deprived and privileged profiles within each of the 22 metropolitan areas, according to COVID-19 vaccination rate.

**Results:**

Testing rates were lower in socioeconomically deprived metropolitan areas than in privileged ones, except during wave 4 (July-October-2021), when testing rates were more similar. Incidence rates were higher in deprived areas (waves 2–4, July-2020 to October-2021), but this pattern reversed between waves 6–9 (March-2022 to March-2023). Meta-regressions indicated that high vaccination coverage was associated with a narrower gap in testing between deprived and privileged areas. Moreover, for each metropolitan area, the higher the level of deprivation in a zone within the deprived profile, the greater the deprived-privileged gap in under-testing.

**Conclusions:**

The impact of socioeconomic inequalities on testing and incidence patterns during the COVID-19 pandemic in each metropolitan area in France was driven by the most deprived zones; this impact varied across epidemic waves. Higher vaccination rates and government health measures (lockdowns, mandatory health pass) may have reduced this variation.

## Introduction

Many studies have highlighted that socioeconomic inequalities were exacerbated during the COVID-19 pandemic [1–6]. This is also true in France, where socioeconomically deprived people were the most severely impacted population, as demonstrated by the Epicov survey [7] and a Santé Publique France study [8]. National-level French studies have analyzed socioeconomic health inequalities related to the pandemic at various geographical levels, and found disparities at the inter-departmental (i.e., sub-regional administrative level) [9–12], and the finer IRIS (sub-municipal level) scales [13].

However, these did not always consider spatial heterogeneity and specific local (i.e., metropolitan area level) geographical contexts related to socioeconomic and pandemic-related factors. Accordingly, their results cannot be extrapolated to all areas of metropolitan France. Moreover, deprivation indices commonly used to study socioeconomic inequalities in health in France (e.g., French Deprivation Index (FDEP), European Deprivation Index (EDI)) do not always fully capture local socioeconomic disparities. This is especially true for studies which divide deprivation indicators into national quintiles. For example, the EDI indicator ‘car ownership’ may have a different impact in urban and rural areas [13,14].

It is clear that, as for any disease, the spread of the COVID-19 pandemic varied according to the level of viral circulation; however, it is possible that the timing and implementation of local protective measures also impacted how it spread. France adopted the ‘test, trace, and isolate’ strategy in May 2020, which was offered free of charge until October 2021, coinciding with the end of wave 4. The first national lockdown was implemented between March and May 2020, while the second and third (from October to December 2020, and April to June 2021, respectively) were less stringent. The vaccination strategy was implemented in five stages from January 2021 onward, with vaccination provided free of charge. Local protective measures differed according to the administrative area: for example, with regard to the second COVID-19 lockdown, the Alpes-Maritimes department was the first to introduce it (weekends only) at the end of February 2021; other departments quickly followed suit, introducing lockdowns in mid-March. This was extended to the rest of France between April and May to become the second national lockdown. Healthcare interventions also differed according to context; for example, health mediator outreach teams were mobilized in socially deprived areas of Marseille but not in other smaller metropolitan areas [15]. It is possible that disparities in population demographics and the spatial and chronological variations in implemented health protection measures across different geographical areas (e.g., sub-departmental, departmental, or regional) may have contributed to the heterogeneous patterns of COVID-19 incidence and testing observed in France over the period of the pandemic.

Using different methods, a small number of ecological studies focusing on IRIS in the Provence-Alpes-Côte-d’Azur region, in the Alpes-Maritimes department [16,17], and in the various municipalities of Paris [18], confirmed that, in terms of socioeconomic inequalities, deprived areas were more negatively affected by the pandemic than privileged ones. Moreover, the Provence-Alpes-Côte-d’Azur study found lower testing rates in deprived areas (versus privileged areas), suggesting more marked socioeconomic disparities than those reported in national-level studies [4,8,13].

By adopting a comparative IRIS-level approach and accounting for the specific socioeconomic profiles of each of France’s 22 metropolitan areas, this study aimed to analyze variations in testing and incidence rates across these areas for each of the country’s epidemic waves. Besides providing a better understanding of territorial health inequalities in France during the pandemic, this analysis provides insights for public health preparedness and local policy design in the face of a future pandemic. Moreover, this is the first study to compare socioeconomic inequalities in COVID-19 testing and incidence across multiple French metropolitan areas at the IRIS level.

## Materials and methods

### Ethics statement

Authorization to conduct this study was granted by the Ethics Committee of Aix-Marseille University (approval number 2022-10-20-006) on 20 October 2022. The study is based on aggregated data (at the IRIS spatial scale) from routine surveillance related to COVID-19 testing results, provided by Santé publique France (SpF), the French national public health agency and regulatory authority. The use of anonymized routine surveillance data for research purposes does not require informed consent from the individuals concerned. As no individual-level data were accessed or used in this study, full anonymity and compliance with all relevant ethical and legal standards were ensured. The raw COVID-19 data aggregated at the IRIS spatial level were obtained from the French national public health agency (Santé Publique France) and are not publicly available. Access to these data requires prior approval from Santé Publique France and may be granted upon reasonable request to prada@santepubliquefrance.fr or through this website: https://www.santepubliquefrance.fr/nous-contacter/acces-aux-documents-administratifs-et-aux-donnees-scientifiques2. All other data used in this study are within the manuscript and its Supporting Information files. The data were stored securely for a period of three years after the end of the study, on the SESSTIM research unit’s (Aix Marseille University) encrypted servers.

### Study design

**Fig 1:**
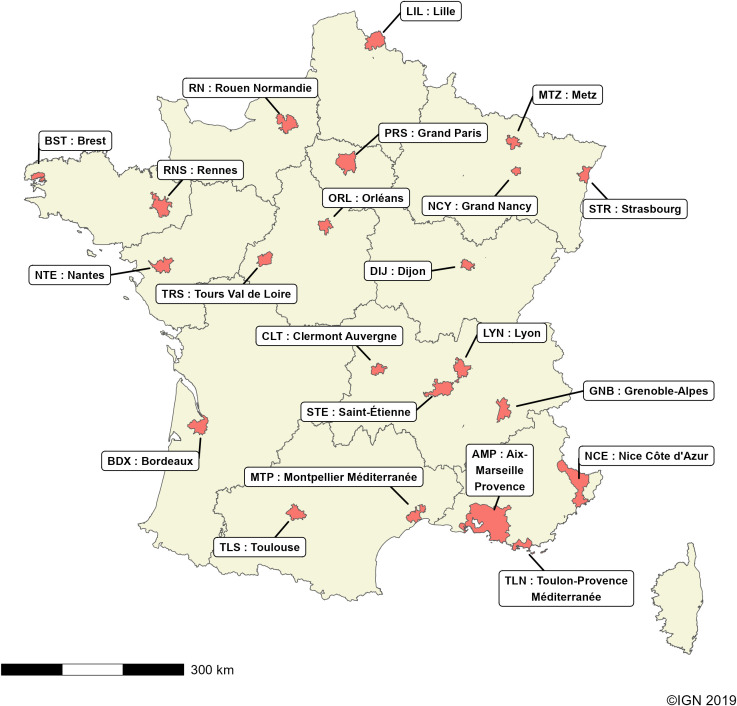
Localization of the 22 French metropolitan areas.

We conducted an ecological study of the COVID-19 pandemic across all of France’s 22 metropolitan areas at the IRIS spatial scale (see above). A total of 7756 IRIS were included (Fig 1).

### Study period

We used data on the number of COVID-19 tests and diagnosed positive cases at the IRIS level. The data were divided into nine epidemic waves. These waves differed in terms of testing and disease incidence levels, as well as in terms of the protective health measures implemented (e.g., lockdowns, health pass, vaccination). The start and end dates for the nine waves were identified as follows:

- For waves 1–3, we used the dates determined by the French National Institute of Statistics and Economic Studies (INSEE) [15]. As we did not have complete data for the first wave, we excluded them and retained data only from the start date of the second wave (6 July 2020) onward.
- To identify subsequent waves, we examined the daily incidence rate, which we smoothed using a 7-day rolling mean (S1 Data). A new wave was considered to begin when the incidence rate increased for at least 14 consecutive days, and to end when it decreased for at least 14 consecutive days. With this method, six additional waves were identified.

Therefore, there were a total of eight analysis periods (from 6 July 2020–4 March 2023); we aggregated the number of tests and diagnosed cases per wave. The same method was applied to new hospitalization cases (Fig 1 in S1 Text).

### Variables

Data sources are described in the appendix (Table 1 in S1 Text).

#### COVID-19 data.

The numbers of COVID-19 tests and cases on a rolling 7-day basis, per IRIS, were provided by Santé Publique France (the French National Public Health Agency) through the SI-DEP information system, which collects and aggregates information about COVID-19 testing and diagnosis. We used France’s daily incidence rate from the SI-DEP system, which is available on open access.

Daily data describing protective measures in France were available from the Oxford COVID-19 Government Response Tracker, where higher index values corresponded to more severe and numerous policy measures (S1 Data) [19].

Weekly data on vaccination rates were available from the French Health Insurance System at the metropolitan area scale (S2 Data).

#### Socioeconomic profiles.

**Fig 2:**
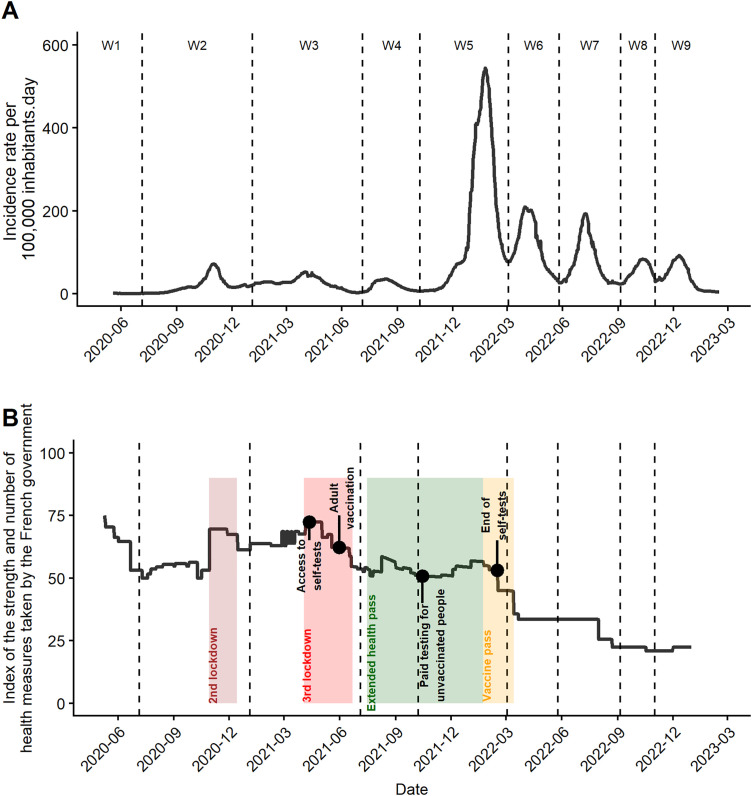
Daily evolution of the COVID-19 incidence rate (A) and of the government’s protective health measures (B). (A) The curve represents a 7-day moving average of the incidence rate. (B) The index (varying from 0 to 100) corresponds to the accumulation and rigour of the government’s health measures, according to the Oxford index in France. The dotted lines correspond to the boundaries of the epidemic waves from wave 2 (W2) to wave 9 (W9).

We used socioeconomic data at the IRIS level from INSEE to create socioeconomic profiles based on the 2017 national census and on an INSEE-developed statistical system to measure social and fiscal localized income (called Filosofi). Socioeconomic variables were selected based on indicators from the following dimensions: family and household; immigration and mobility; education; housing; employment and income. This selection enabled us to create an indicator in line with the method described by Lalloué [20]. We used the 2017 European Deprivation Index (EDI) at the IRIS level, which we requested from MapInMed [21]. Detailed information for each variable is provided in the supplementary information section (S3 Data; Table 2 in S1 Text). For each metropolitan area, we used these quantitative variables to perform a principal component analysis, followed by hierarchical ascending clustering to build socioeconomic profiles [9]. Maps of these profiles and histograms of the EDI,coloured according to each profile,can be consulted for all 22 French metropolitan areas in the appendix (Fig 2AB–23AB in S1 Text). For all the socioeconomic profiles created for each metropolitan area, we computed the median EDI and identified the most and the least socioeconomically deprived profiles.

#### Adjustment variables.

We used several variables to adjust for testing and incidence rates. Testing indicators included the annual number of general practitioner consultations per inhabitant at the municipality level, as well as the number of laboratories and pharmacies, and the number of retirement homes at the IRIS level. For each IRIS, we also computed the log population density adjusted for residential area, a composite indicator describing age structure and environmental characteristics. Further details are provided in the appendix (‘Adjustment variables’ section in S1 Text).

### IRIS selection

First, we excluded activity-based and mixed activity-residential IRIS to retain only residential IRIS. Second, we excluded IRIS with fewer than 30 households, as many variables focused on household and housing characteristics. IRIS with missing data on the median income were also excluded, except when these data were available at the municipal scale. Furthermore, we excluded IRIS where the number of positive cases exceeded the population during several 7-day rolling periods (likely due to misallocation of test results). Finally, one IRIS in the Nice metropolitan area, which we categorized as deprived, had a particularly high testing rate; we excluded it because the strong leverage effect it exerted in the multivariate testing models could have skewed results.

### Statistical analyses

Statistical analyses, described as graphical and cartographic representations, were performed using R software (version 4.2.2, R Development Core Team, R Foundation for Statistical Computing, Vienna, Austria).

#### Trend analysis.

For each epidemic wave, we plotted the relationship between the median EDI of the most deprived IRIS in each metropolitan area and the IRIS’s testing, incidence, and positivity rates (S4 Data). Locally estimated scatterplot smoothing (LOESS) curves with 95% confidence intervals were fitted to characterize the association between deprivation and epidemic indicators among the most deprived profile. For each epidemic wave, we classified metropolitan areas according to the position of their most deprived profile relative to the LOESS curve and its confidence intervals (CI) (i.e., below the CI, within the CI and below the curve, within the CI and above the curve, and above the CI). These classifications were tracked across waves using heatmaps and Sankey diagrams to visualize their temporal changes and to identify persistent or evolving patterns in epidemic dynamics among the most deprived profile (Fig 24AB–25AB in S1 Text). All these plots and diagrams were plotted on positivity rates (Fig 26ABC in S1 Text). Finally, we compared the cumulative median vaccination coverage for each wave at the metropolitan area level with the median EDI and testing rates of the most deprived profile to assess whether socioeconomic inequalities observed in testing were also linked to vaccination coverage (S4 Data).

#### Statistical model.

We used generalized additive mixed models (GAMMs) to quantify differences in testing and incidence rates between the least and the most deprived profiles (S5 Data). We ran one GAMM for each wave and each metropolitan area. We used a negative binomial distribution to account for data overdispersion, which is appropriate when dealing with count data, and we included the logarithm of the population as an offset. The main variable of interest was socioeconomic profile, with the reference category being the least socioeconomically deprived profile. Incidence rate models included the logarithm of the number of tests per person per IRIS. We included a random effect at the municipality level to account for similarities among IRIS within the same municipality. We used a random Markov field, which allowed us to account for the spatial autocorrelation of IRIS based on the Queen’s criterion of contiguity. We performed sensitivity analyses by running models with and without the adjustment variables (S5 Data; Fig 27AB in S1 Text). For the Nice metropolitan area, we ran univariate and multivariate models on the testing rates with and without the outlier IRIS with the particularly high testing rate (see above). (Table 3 in S1 Text).

Below, we describe the model for the testing rates (including spatial autocorrelation and random effect):


$$\begin{array}{cc} log(number\;of\;tests\;in\;wavei)=\; & {offset\left(log(population)\right)}_{IRIS}+{socioeconomic\;profiles}_{ref:the\;least\;deprived\;(IRIS)}\;\\ & +adjustment\;variables \end{array}$$

And the model for the incidence rate (including spatial autocorrelation and random effect) was as follows:


$$\begin{array}{cc} log(number\;of\;cases\;in\;\;wavei)=\; & {offset\left(log(population)\right)}_{IRIS}\;\\ & +\;{\text{log}(number\;of\;tests\;per\;person\;in\;\;wavei)}_{IRIS}\\ & +{socioeconomic\;profiles}_{ref:the\;least\;deprived\;(IRIS)}\;\\ & +adjustment\;variables \end{array}$$

#### Meta-regression on adjusted testing rate ratios (aTRRs) and adjusted incidence rate ratios (aIRRs) of the most deprived socioeconomic profile.

Results from the previous models were used in linear meta-regressions to explain aTRRs and aIRRs (S6 Data). We included three covariates as linear predictors: wave number, the median cumulative vaccination rate during each wave in each metropolitan area, and the median EDI of the most deprived profile in each metropolitan area. We plotted the mean aTRRs and aIRRs by wave. For the sensitivity analyses, we ran linear meta-regressions using TRRs and IRRs obtained from univariate models (Fig 28AB–29AB in S1 Text).


$$\begin{array}{cc} og(aTRRs,\;aIRRS)=\; & wave\;+\;median\;cumulative\;vaccination\;rate\;(\%)\;+\;median\;EDI\;of\;the\;most\;\\ & deprived\;socioeconomic\;profile\;in\;each\;metropolitan\;area \end{array}$$

## Results

### IRIS selection

Of the 7756 IRIS in the 22 metropolitan areas, 7186 were included in our analysis. We excluded 554 non-residential IRIS, 6 with fewer than 30 households, and 8 with missing median income data (Table 1). In Nantes, one IRIS reported more cases than inhabitants across several 7-day rolling periods during wave 5 (specifically during the rolling weeks from 12 January 2022 to 29 January 2022). As mentioned above, we excluded one deprived IRIS in Nice because of its leverage effect on testing rates in the multivariate models (Table 1). For these models only, 7 IRIS were excluded for waves 7,8 and 9 due to missing testing and incidence data for these specific periods. These included 1 IRIS in Toulouse, 3 in Grand Paris and 3 in Lyon.

**Table 1:** Number of IRIS^a^ for each French metropolitan area after each selection step.

	Number of IRIS after each step					
Metropolitan area	Step 1: Initial number of IRIS	Step 2: Exclusion of activity-based and mixed activity/residential-based IRIS	Step 3: Exclusion of IRIS with fewer than 30 households	Step 4: Exclusion of IRIS where median income data was unavailable	Step 5: Exclusion of IRIS where the number of cases exceeded the number of inhabitants	Step 6: Exclusion of one deprived IRIS in Nice with unusually high testing rate, due to its leveraging effect on multivariate models for testing rates.
**Bordeaux**	277	259*	259	259	259	259
**Brest**	93	80*	80	80	80	80
**Clermont Auvergne**	103	92*	92	92	92	92
**Dijon**	122	108*	108	107*	107	107
**Strasbourg**	195	174*	174	174	174	174
**Grenoble-Alpes**	201	184*	184	183*	183	183
**Aix-Marseille**	779	705*	704*	704	704	704
**Lyon**	512	467*	467	467	467	467
**Grand Nancy**	115	102*	102	102	102	102
**Grand Paris**	2841	2673*	2672*	2672	2672	2672
**Lille**	509	478*	476*	476	476	476
**Nice Côte d’Azur**	236	230*	230	224*	224	223*
**Rouen Normandie**	241	218*	217*	217	217	217
**Toulon-Provence-Méditerranée**	176	168*	167*	167	167	167
**Metz**	113	106*	106	106	106	106
**Montpellier Méditerranée**	160	144*	144	144	144	144
**Nantes**	234	206*	206	206	205*	205
**Orléans**	117	110*	110	110	110	110
**Rennes**	172	158*	158	158	158	158
**Saint-Etienne**	187	185*	185	185	185	185
**Toulouse**	253	237*	237	237	237	237
**Tours**	120	118*	118	118	118	118
**Total**	7756	7202	7196	7188	7187	7186

*Indication of a decrease in the total number of IRIS selected compared to the previous step.

^a^IRIS: a spatial unit typically representing neighborhoods of approximately 2,000 residents, which is relatively homogeneous in terms of socioeconomic characteristics [22]).

### Description of epidemic waves

The eight analysed epidemic waves had different incidence rates, waves 5–9 having higher incidence rates than the earlier waves (Fig 2A). Higher index values (see Government Response Tracker above) characterized waves 1–3 due to lockdowns and curfews (Fig 2B). The beginning of wave 3 (January 2021) coincided with the start of the vaccination strategy for people aged over 75, which was extended to people aged over 18 in May 2021. Self-tests were also introduced during this wave (Fig 2B). Subsequently, the index decreased during waves 4 and 5 with the introduction of a health pass, which was later converted into a vaccination pass (Fig 2B). The start of wave 5 corresponded to the introduction of fees for tests among unvaccinated populations (Fig 2B). A fall in the index was observed during waves 6–9, corresponding to fewer protective health measures (Fig 2B).

**Fig 3:**
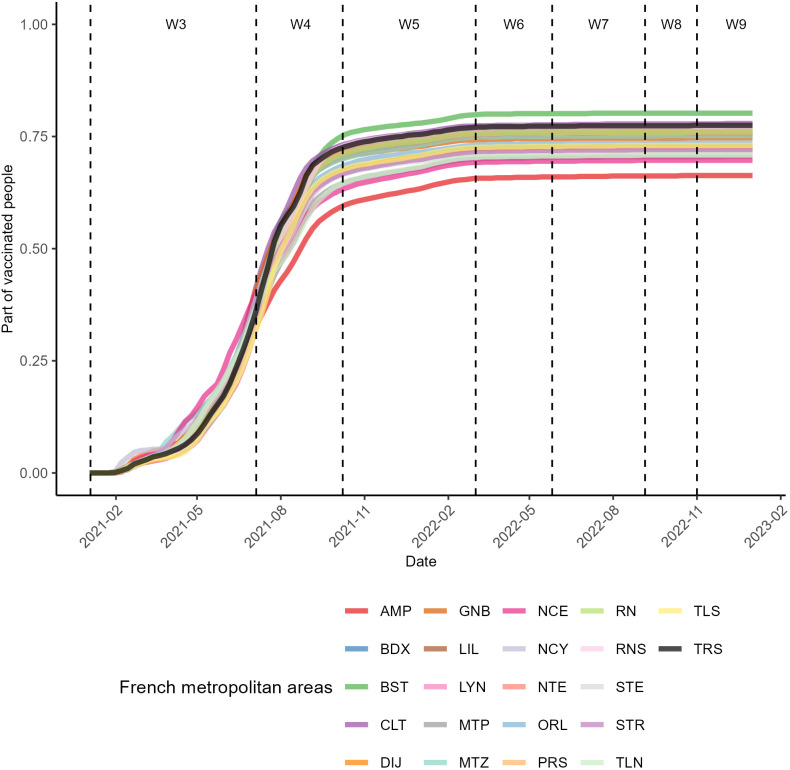
Daily evolution of the COVID-19 vaccination rate across the 22 French metropolitan areas.

Vaccination steadily increased after its introduction in wave 3 (Fig 3). However, this increase was only slight in wave 5 and stabilized from wave 6 onwards (Fig 3).

### Testing and incidence rates for the most socioeconomically deprived profile according to metropolitan area and epidemic wave

**Fig 4:**
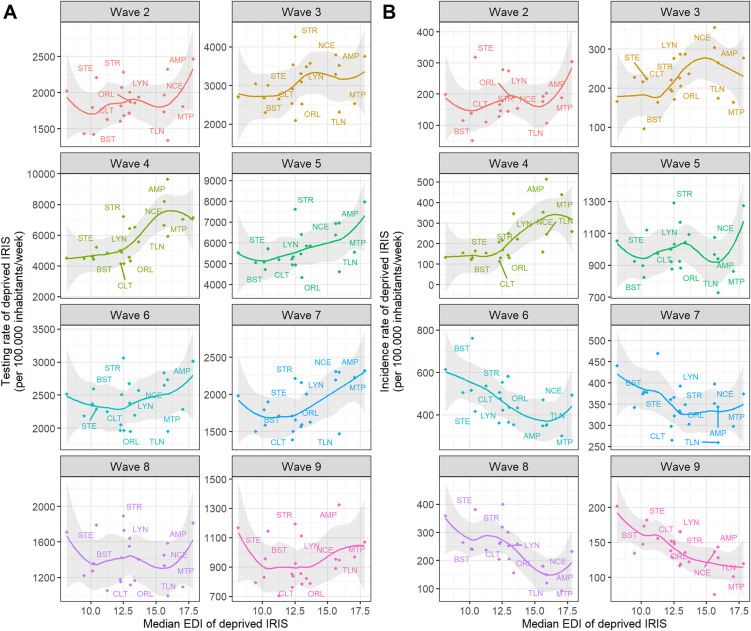
(A) Testing and (B) incidence rates of the most deprived profile in each of the 22 French metropolitan areas according to epidemic wave (waves 2 to 9). **(A)** Testing rates and **(B)** incidence rates were computed only for IRIS belonging to the most deprived profile in each metropolitan area. The associated median EDI^a^ values for all 22 French metropolitan areas were then calculated. Values for each metropolitan area were then compared with these median figures. Graphs show smoothed LOESS curves with 95% confidence intervals shaded in grey. AMP: Aix-Marseille Provence, BST: Brest, CLT: Clermont, LYN: Lyon, MTP: Montpellier, NCE: Nice, ORL: Orléans, STE: Saint-Étienne, STR: Strasbourg, TLN: Toulon. ^a^EDI: European deprivation index, ^b^LOESS: locally estimated scatterplot smoothing.

During wave 4, testing rates were higher in the most deprived profiles with higher median EDI values (Fig 4A). A similar trend was observed during waves 5 and 7 (Fig 4A). Heatmaps showed that the most deprived profil in some metropolitan areas had either consistently high testing rates (Strasbourg (STR), AMP, Lyon (LYN)) or consistently low rates (Toulon (TLN), Orléans (ORL), Montpellier (MTP) and Clermont (CLT)) for at least four waves compared to what was expected given their median EDI values (Fig 24AB in S1 Text).

Incidence rates were higher in the most deprived profiles with higher median EDI values (Fig 4B) during waves 3 and 4, but were lower from wave 6 onward (Fig 4B). Positivity rates followed similar trends (Fig 26A in S1 Text). Heatmaps revealed persistently high incidence rates across at least 4 waves in some metropolitan areas (Nice (NCE), Lyon (LYN), Saint-Étienne (STE), and Strasbourg (STR)), while others showed consistently lower incidence rates (Montpellier (MTP), Toulon (TLN), and Orléans (ORL)) (Fig 25AB in S1 Text).

### Vaccination rates according to French metropolitan area and epidemic wave

**Fig 5:**
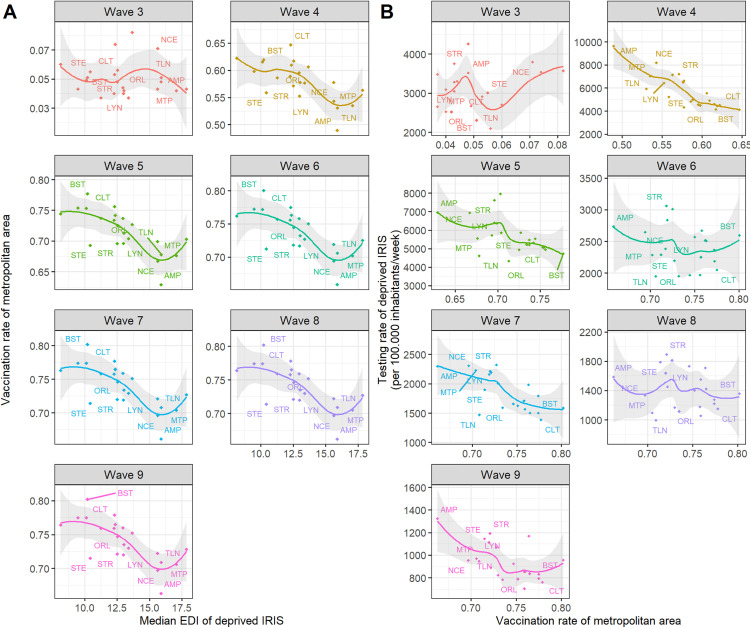
Median vaccination rates across the 22 French metropolitan areas and epidemic waves (waves 3 to 9). Median vaccination rates across metropolitan areas (A) were compared to the median EDI^a^ (B) and to IRIS-level testing rates belonging to the most deprived profile across all 22 metropolitan areas. Graphs show smoothed LOESS curves with 95% confidence intervals shaded in grey. AMP: Aix-Marseille Provence, BST: Brest, CLT: Clermont, LYN: Lyon, MTP: Montpellier, NCE: Nice, ORL: Orléans, STE: Saint-Étienne, STR: Strasbourg, TLN: Toulon. ^a^EDI: European deprivation index, LOESS: locally estimated scatterplot smoothing.

At the metropolitan area level, low vaccination rates were associated with low median EDI values in the most deprived profiles, except during wave 3 (Fig 5A). During wave 4, metropolitan areas with low vaccination rates had higher testing rates for the most deprived profiles (Fig 5B). Similar trends were observed during waves 5, 7 and 9 (Fig 5B).

### Comparison of differences in testing and incidence rates between the most deprived and least deprived socioeconomic profiles across all 22 French metropolitan areas according to epidemic wave

#### Adjusted testing rate ratio of the most deprived profile (aTRRs).

**Fig 6:**
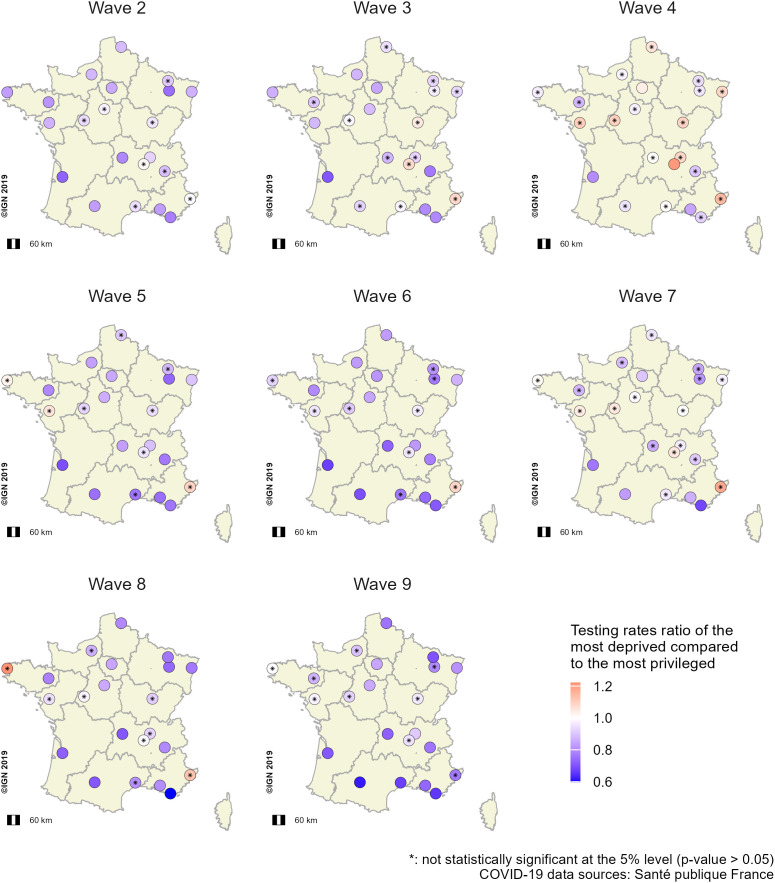
Spatial distribution of aTRRs^a^ between the most deprived and the least deprived profiles across the 22 French metropolitan areas and epidemic waves (waves 2 to 9). The ratios were computed from multivariate GAMMs per wave, comparing testing rates for each metropolitan area between the socioeconomic profile with the lowest median EDI^c^ (i.e., the least deprived) and the other profiles. ^a^aTRRS: adjusted testing rate ratios; ^b^GAMMs: Generalized additive mixed models; ^c^EDI: European deprivation index.

Testing rates were lower in the most deprived IRIS than in the least deprived ones (Fig 6). Differences in aTRRs were observed for the same metropolitan area across the eight waves analyzed (e.g., Toulouse, wave 2: aTRR = 0.83, 95% confidence interval (95% CI) = [0.71 0.97]; wave 9: aTRR = 0.62, [0.51 0.75]) (Fig 6). During wave 4, no significant differences were observed among aTRRs for 18 metropolitan areas, all values being close to 1 (Fig 6). Furthermore, we observed disparities between different metropolitan areas for the same wave (e.g., wave 8, Toulon: aTRR = 0.60, [0.44–0.82], Grand Paris: aTRR = 0.85 [0.81–0.88]) (Fig 6). Unadjusted testing rate ratios (TRRs) showed similar trends (Fig 27A in S1 Text).

#### Adjusted incidence rate ratio of the most deprived profile (aIRRs).

**Fig 7:**
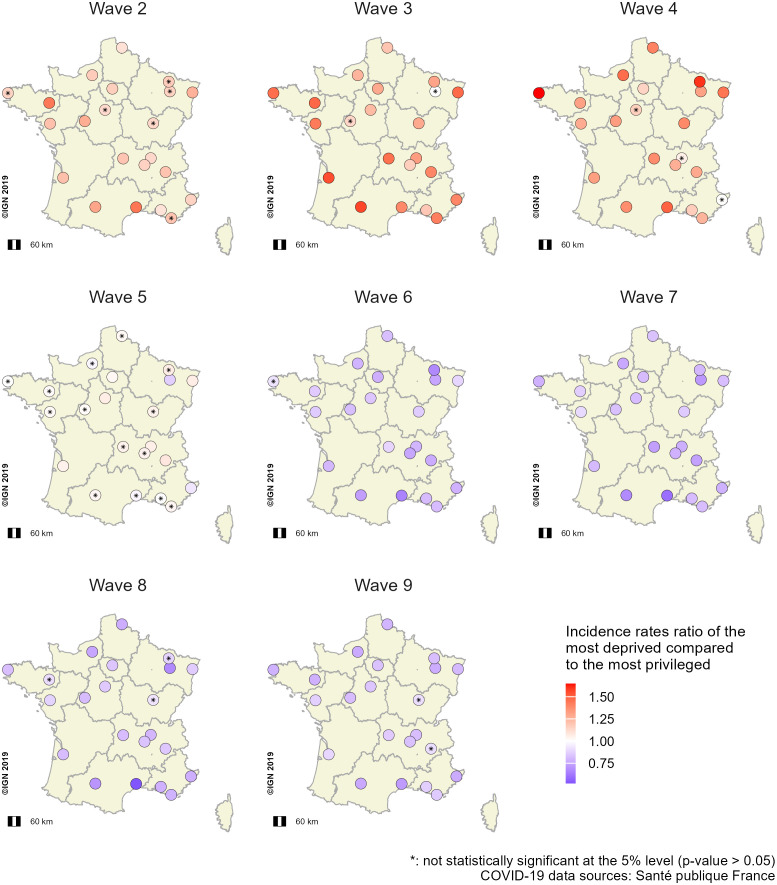
Spatial distribution of aIRRs^a^ between the most deprived and the least deprived profiles across the 22 French metropolitan areas and waves (waves 2 to 9). The ratios were computed from multivariate GAMMs^b^ per wave, comparing incidence rates for each metropolitan area between the socioeconomic profile with the lowest median EDI^c^ (the least deprived IRIS) and the other profiles. ^a^aIRRS: adjusted incidence rate ratios; ^b^GAMMs: Generalized additive mixed models; ^c^EDI: European deprivation index.

The disease incidence results can be summarized into three phases. First, from waves 2–4, aIRRs were greater than 1 for all 22 metropolitan areas, indicating that incidence rates were highest in the most deprived IRIS (Fig 7). Second, during wave 5, aIRRs were close to 1 for most metropolitan areas (Fig 7). Finally, from waves 6–9, incidence rates were lower in the most deprived profiles compared to the least deprived one (aIRRs < 1) (Fig 7). Unadjusted incidence rate ratios (IRRs) showed similar trends (Fig 27B in S1 Text).

### Meta-regressions on adjusted testing rate ratios (aTRRs) and incidence rate ratios (aIRRs) of the most socioeconomic-deprived profiles for all metropolitan areas

#### Factors associated with adjusted testing rate ratios (aTRRs).

**Fig 8:**
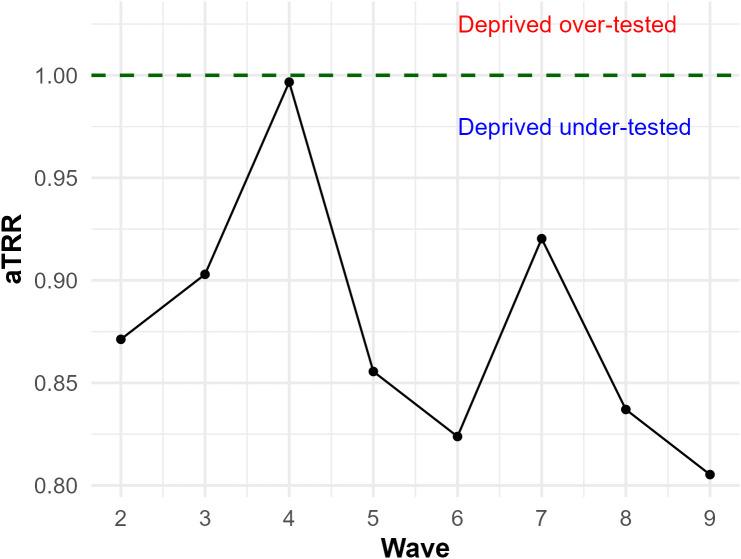
Mean aTRRs^a^ across epidemic waves. aTRRs^a^ were estimated by comparing the most deprived and the least deprived IRIS across each of the 22 French metropolitan areas and each of the eight analyzed epidemic waves using multivariate GAMMs^b^. Green dotted lines correspond to the testing equality point between the least deprived and the most deprived profiles aTRR = 1). ^a^aTRRS: adjusted testing rate ratios. ^b^GAMMs: generalized additive mixed models.

Mean aTRRs were lower than 0.92 (indicating undertesting in the most deprived profiles) for all analyzed waves except wave 4 (mean aTRRs = 1) (Fig 8). Generally, aTRRs decreased across waves, but increased between waves 3 (mean aTRRs = 0.9) and 4 and between waves 6 (mean aTRRs = 0.82) and 7 (mean aTRRs = 0.92) (Fig 8).

Meta-regressions identified three significant predictors of aTRRs. First, aTRRs decreased across successive waves (β = –0.025) (Table 2). Second, higher cumulative vaccination rates (β = 0.0011) and lower EDI values (β = –0.010) were associated with higher aTRRs (Table 2). At the metropolitan area level, when aTRRs were below 1, the higher the median EDI, the greater the deprived-privileged gap in under-testing among the most deprived profiles. Moreover, high vaccination coverage was associated with a narrower gap in testing between deprived and privileged areas. These results were similar when using unadjusted TRRs (Fig 28AB in S1 Text).

**Table 2:** Results of multivariate meta-regression on the logarithm of aTRRs^a^.

Variables	Estimate	95% CI^b^	p-value
**Wave**	−0.025	[−0.039, −0.011]	< 0.001
**Median cumulative vaccination rate (%)**	0.0011	[0.0001, 0.0021]	0.034
**Median EDI**^**c**^ **of the most deprived profile**	−0.010	[−0.019, −0.005]	0.001

^a^TRRs^a^ were estimated by comparing the most deprived and the least deprived IRIS across each of the 22 French metropolitan areas and each of the eight analyzed epidemic waves using multivariate GAMMs^d^. The table shows estimated betas for the three IRIS significant predictors (i.e., explanatory variables) (wave, median cumulative vaccination rate and the most deprived profile’s median EDI) with confidence intervals (95% CI) and p-values. ^a^aTRRs: adjusted testing rate ratios; ^b^CI: confidence interval; ^c^EDI: European deprivation index; ^d^GAMMs: generalized additive mixed models.

#### Factors associated with adjusted incidence rate ratios (aTRRs).

**Fig 9:**
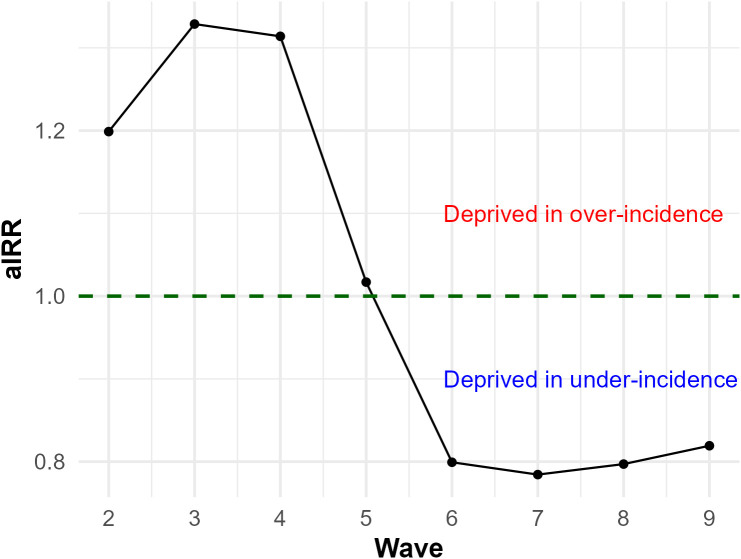
Mean aIRRs^a^ across all eight epidemic waves analyzed. aIRRs^a^ were estimated by comparing the most deprived and the least deprived profiles across each of France’s 22 metropolitan areas for eight of the country’s nine epidemic waves using multivariate GAMMs^b^. Green dotted lines correspond to the testing equality point between the least deprived and the most deprived profiles (aIRR = 1). ^a^aIRRS: adjustment incidence rate ratios; ^b^GAMMs: generalized additive mixed models.

Mean aIRRs during waves 2,3, and 4 were higher than 1.2 (indicating over-incidence in the most deprived profiles), respectively (Fig 9). During wave 5, aIRRs were close to 1 (mean aIRRs = 1.02) (Fig 9). From waves 6–9, they were lower than 0.82 (indicating under-incidence in the most deprived profiles) (Fig 9).

Meta-regressions showed a significant decrease in aIRRs over waves (β = –0.07) (Table 3). aIRRs were negatively associated with both cumulative vaccination rates (β = –0.0013) and median EDI values (β = –0.01) (Table 3). The variables were associated with incidence differently depending on the baseline: when IRR > 1, they reduced the excess incidence in deprived profiles, while when IRR < 1, they further lowered their incidence, enhancing their relative advantage. Meta-regressions using unadjusted incidence rate ratios (IRRs) confirmed the wave effects, with consistent directional associations for vaccination and EDI, although these were not statistically significant (Fig 29AB in S1 Text).

**Table 3:** Results of multivariate meta-regression on the logarithm of aIRRs^a^.

Variables	Estimate	95% CI^b^	p-value
**Wave**	−0.066	[−0.082, −0.051]	< 0.001
**Median cumulative vaccination rate (%)**	−0.0013	[−0.0025, −0.0002]	0.025
**Median EDI**^**c**^ **of the most deprived profile**	−0.010	[−0.018, −0.002]	0.019

^a^IRRs^a^ were estimated by comparing the most deprived and the least deprived IRIS across each of France’s 22 metropolitan areas and each of the country’s 8 epidemic waves using multivariate GAMMs^d^. The table shows estimated betas for the three IRIS significant predictors (i.e., explanatory variables) (wave, median cumulative vaccination rate and median EDI of deprived profile) with confidence intervals (95% CI) and p-values. ^a^aIRRs: adjustment incidence rate ratios; ^b^CI: confidence interval; ^c^EDI: European deprivation index; ^d^GAMMs: generalized additive mixed models.

## Discussion

This study examined the relationship between socioeconomic profiles and both COVID-19 testing and incidence patterns across France’s 22 metropolitan areas and eight of the country’s eight epidemic waves. By constructing local socioeconomic profiles, we gained insights into the specific context of each metropolitan area.

Testing and incidence rates among IRIS belonging to the most deprived profile varied not only from one metropolitan area to another, but also according to epidemic wave (waves 2–9). Overall, testing in these IRIS was lower than in the least deprived areas, except during wave 4. In terms of incidence, the most deprived IRIS were more affected during waves 2–4, but were the least affected during waves 6–9. Contrasts in testing and incidence rate ratios were observed across all metropolitan areas and across all analyzed epidemic waves. Meta-regressions suggested that vaccination rates in metropolitan areas, epidemic wave, and higher deprivation (median EDI value) among the most deprived IRIS were associated with these contrasts.

The implementation of specific health policies may partly explain the contrasts observed across epidemic waves and metropolitan areas. During wave 4, more homogeneous testing between the least deprived and the most deprived IRIS may be associated with the introduction of an obligatory health pass, which required people to be vaccinated or to have a recent negative test if they wished to access public places and the workplace. We hypothesized that this policy may have temporarily increased testing in the most deprived IRIS. Meta-regression analyses also suggested that vaccination coverage at the metropolitan level was associated with a narrower deprived-privileged gap in under-testing in the most deprived IRIS, as observed during wave 4. Previous studies have reported higher vaccination uptake among more privileged populations [23–25]. We hypothesized that vaccination coverage was higher in the least deprived IRIS than in the most deprived IRIS, and that this difference, together with the introduction of the health pass, may partly explain the narrower gap in under-testing in the most deprived IRIS. Heterogeneity was observed between metropolitan areas, with some showing higher testing among the most deprived IRIS, while others showed lower testing, highlighting the importance of local contextual factors. From wave 5 onward, the decline in testing among the most deprived IRIS compared with the least deprived IRIS coincided with the introduction of fee-based testing for unvaccinated individuals. In line with previous studies on financial barriers to healthcare access in deprived populations [26,27], we hypothesized that this policy change may be associated with the observed testing patterns.

Contrasts in incidence rates across metropolitan areas and waves could be explained by different geographical health policies, including different types and timing of lockdown during waves 2 and 3, and the introduction of vaccination at the start of wave 3. The most deprived IRIS were the most infected during waves 3 and 4, which coincided with the start of vaccination. However, vaccination may have had a reduced protective effect against the Omicron variant (from wave 5 onward) [28], which was more contagious than previous variants [29]. From wave 6 onward, a reversal trend, where the least deprived IRIS exhibited higher incidence rates, was correlated with the lifting of government protective health policies. A cross-sectional survey suggested that the least deprived populations may have been better protected against infection when lockdowns were in place [30]. This may explain the under-incidence observed in the least deprived IRIS during the initial waves (waves 2–4). After restrictions were lifted from wave 6 onward, we hypothesize that social habits, such as more frequent mobility, leisure activities, and social gatherings of inhabitants living in the least deprived IRIS were associated with increased exposure to infection. Finally, we observed that the reduction in over-incidence and the increase in under-incidence among the most deprived profiles (compared to the least deprived) tended to be associated with their higher level of deprivation. This trend is also reflected in our graphs, with incidence and positivity rates declining from wave 6 onwards among the most deprived profiles with the highest degree of deprivation. One possible explanation is that health mediation initiatives, such as those implemented in Marseille [15], primarily targeted the most socioeconomically deprived areas; cities implementing such initiatives may also be those concentrating the most deprived areas across metropolitan areas.

Several study limitations must be acknowledged. First, the statistical models may have been affected by multicollinearity between socioeconomic variables and population density. Nonetheless, observed trends were consistent across all 22 metropolitan areas and the eight studied epidemic waves. They were also consistent with the sensitivity analyses. Second, there may also be biases linked to the topography of metropolitan areas such as Nice, where the smaller differences in testing rates between the most deprived and the least deprived profiles may be due to the presence of mountainous and rural areas to the north of the area, where access to testing is more difficult. This point has already been raised in a previous study of the Provence-Alpes-Côte d’Azur region, where Nice is located [13]. This finding underlines the importance of choosing appropriate spatial scales and study areas when measuring socioeconomic inequalities in health, and the need to perform analyses on urban areas. Third, we defined epidemic waves based on incidence, which in turn depends on testing rates. Wave limits were confirmed using hospitalization data, which are independent of testing. Nevertheless, intra-wave variations in incidence and testing may have occurred between the most and the least deprived IRIS. Fourth, we had no access to data on vaccination at the IRIS level, which could have provided additional information on incidence and testing behaviors. A part from the cost of the tests, vaccine hesitancy may also have been due to low health literacy, less confidence in the healthcare system, and fear of stigmatization in the event of a positive test [13,26,31]. Fifth, exploring behavioral factors was not possible due to the study design, where individual determinants could not be taken into account. Using mobility data could provide a better picture of these behaviors during periods when health measures were in place [32,33].

Finally, this study is limited by ecological bias, where results cannot necessarily be applied at the individual scale [3,28]. However, since no individual data on socioeconomic determinants are available in France, studying inequalities in the country’s 22 metropolitan areas at a fine geographical scale (i.e., IRIS level) may have helped reduce this bias by better capturing local socioeconomic contexts and specific pandemic situations. Vandentorren et al. reported that the most deprived IRIS (last EDI quintile) had the lowest testing rates compared to the least deprived IRIS (first EDI quintile) [8]. In our study, we observed that within these most deprived IRIS, higher levels of deprivation were associated with greater under-testing. This suggests that large variations exist even within the most deprived metropolitan areas. These variations may not be captured when deprived IRIS are treated as a single, homogeneous group, for example, in national-level analyses. Moreover, when comparing regional-level results from a previous study on testing in the Provence-Alpes-Côte d’Azur region with our findings for three metropolitan areas located in that same region (Nice, Aix-Marseille, and Toulon), differences emerged [13]. Specifically, we did not observe any significant difference in testing rates between the most and the least deprived IRIS in Nice. In contrast, in Toulon, the highest levels of under-testing were observed (for the most deprived IRIS) compared to the regional study [13]. In terms of incidence in the Provence-Alpes-Côte d’Azur region, similar contrasts were observed during waves 2 and 3. Aix-Marseille had lower over-incidence among the most deprived IRIS, while Toulon had higher over-incidence (compared to the regional study) [34]. Although previous studies generally reported higher COVID-19 incidence among deprived areas (not necessarily metropolitan-level) [4,8,16,34], our results showed that within the most deprived profiles in each of the country’s 22 metropolitan areas, higher deprivation was associated with lower incidence. This is consistent with Marinée et al., who reported geographical differences in the Alpes-Maritimes department (a sub-regional administrative level), where the most deprived IRIS were differentially affected [17]. This inverse relationship warrants further investigation in future studies.

In terms of strengths, this study is the first to compare COVID-19 incidence and testing levels across different local-level (i.e., IRIS) areas in France and over a large period (covering eight of the country’s nine epidemic waves). Our findings suggest that adapting government health measures to local contexts could lead to more effective public health interventions. This could be particularly valuable in future pandemics to reduce health inequalities and limit the disproportionate impact on persons living in the most socioeconomically deprived areas.

## Conclusion

In conclusion, this study highlights the role of socioeconomic inequalities in COVID-19 testing and incidence across the 22 metropolitan areas in European France. Testing and incidence gaps at the metropolitan area level between the most deprived and the least deprived IRIS were associated with the level of deprivation of the former. This suggests an association between these gaps and protective measures such as vaccination rates, lockdowns and the health pass. Furthermore, our findings leading us to recommend strategies tailored to the local context rather than uniform national strategies, as was the case in France during COVID-19 pandemic.

## Supporting information

S1 Text Supplementary materials and methods. (PDF)

S1 Data Index of the level of stringency and the number of protective COVID-19 health measures implemented by the French Government during the pandemic, and incidence rates in all 22 metropolitan areas in European France. (XLSX)

S2 Data COVID-19 vaccination rates for all 22 metropolitan areas in European France. (XLSX)

S3 Data Variables at the IRIS scale. The database contains all socioeconomic variables used to compute the socioeconomic profiles and adjustment variables.
(XLSX)

S4 Data Testing and incidence rates, as well as median EDI values in the most deprived profile for all 22 metropolitan areas in France; median vaccination rates for all 22 areas and according to epidemic wave. (XLSX)

S5 Data Unadjusted and adjusted testing, as well as incidence rate ratios of the most deprived IRIS profile compared to the least deprived IRIS profile in each of the 22 metropolitan areas in European France and according to epidemic wave. Ratios were computed using generalized additive mixed models (GAMMs).
(XLSX)

S6 Data Database used to perform multivariate meta-regressions of unadjusted and adjusted testing and incidence rate ratios. (XLSX)
