# Supplementary material for: Socioeconomic inequalities and the COVID-19 pandemic in France: Territorial analyzes based on epidemic wave and metropolitan area
Source: PLoS One. 2026 May 8;21(5):e0348201. doi: 10.1371/journal.pone.0348201 (PMC13155628; doi:10.1371/journal.pone.0348201)
Supplement: S1 Text — (PDF) [file pone.0348201.s001.pdf]

Appendix

Data sources

Table 1: Data sources

Database name	Source	URL
Census population of 2017 (IRIS scale)	INSEE	https://www.insee.fr/fr/information/4467366
Median income from Filosofi database (2018)	INSEE	https://www.insee.fr/fr/statistiques/5055909 (IRIS scale) https://www.insee.fr/fr/statistiques/5009218 (municipality scale)
European deprivation index (EDI)(2017)	MapinMed	https://anticipe.eu/plateformes/MAPinMED
Equipment database (2021)	INSEE	https://drees2-sgsocialgouv.opendatasoft.com/explore/dataset/530_l-accessibilite-potentielle-localisee-apl/information/.
Localised potential accessibility (LPA)(2021)	DREES	https://drees2-sgsocialgouv.opendatasoft.com/explore/dataset/530_l-accessibilite-potentielle-localisee-apl/information/.
Land cover (2018)	Copernicus	https://land.copernicus.eu/pan-european/corine-land-cover/clc2018?tab=download.
Index of government stringency (2020-2023)	Blavatnik School of Government, Université d'Oxford	https://github.com/OxCGRT/covid-policy-tracker/tree/master/data.
Vaccination COVID-19 (2021-2023)	French health insurance	https://datavaccin-covid.ameli.fr/explore/dataset/donnees-de-vaccination-par-epci/information/.
Incidence rates for Mainland France (2020-2023)	SI-DEP	https://www.data.gouv.fr/fr/datasets/donnees-de-laboratoires-pour-le-depistage-a-compter-du-18-05-2022-si-dep/.
Hospitalization rates for mainland France	SIVIC	https://www.data.gouv.fr/datasets/donnees-hospitalieres-relatives-a-lepidemie-de-covid-19
Shape of 200m tiles (to compute population density)(2017)	INSEE	https://www.insee.fr/fr/statistiques/6215138?sommaire=6215217
Shape of IRIS (2019)	Geoservices	https://geoservices.ign.fr/contoursiris

Breakdown of epidemic waves with data on new hospitalization cases

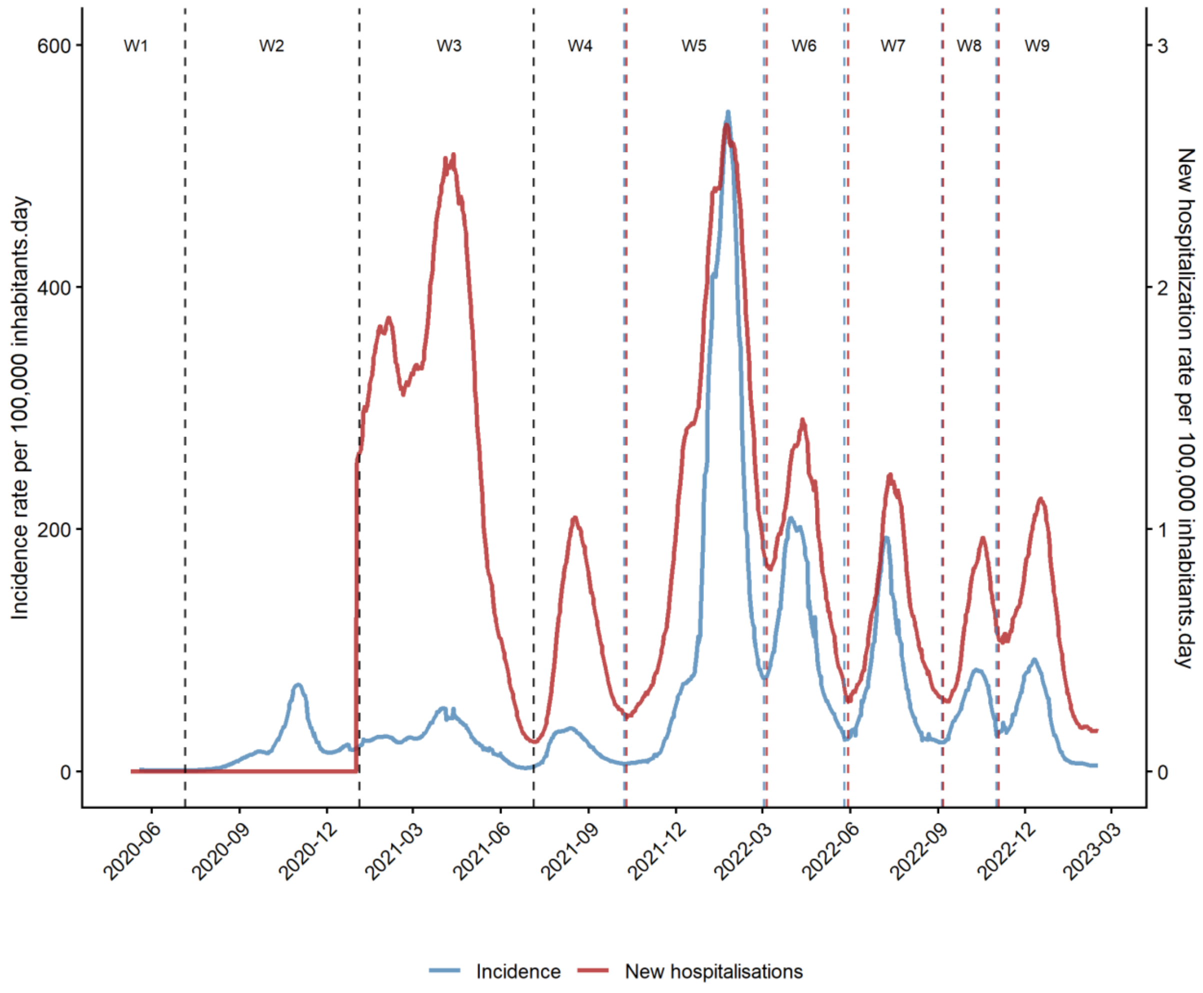

Fig 1: Breakdown of epidemic waves with data on new hospitalizations and incidence. The boundaries for the breakdown using hospital admission data are shown in red, and those for the breakdown using COVID-19 data are shown in blue. Those in black correspond to the breakdown carried out by INSEE [1].

Socioeconomic variables and maps

Table 2: Description of socioeconomic indicators used to create socioeconomic profiles

Variable label	Variable name
Proportion of inhabitants in single-person households among the total household population	FM_men_seul
Average household size	FM_taille_men
Proportion of inhabitants in single-parent families among the total household population	FM_fam_mono
Proportion of inhabitants in couples without children in the total household population	FM_couple_sans_enf
Proportion of inhabitants in couples with children in the total household population	FM_couple_avec_enf
Proportion of married or in civil union in the population	FM_pacse_mariage
Proportion of inhabitants outside of households in the total population	FM_hors_men

Proportion of foreign inhabitants in the total population	IM_etrangers
Proportion of immigrants in the total population	IM_immigrés
Proportion of inhabitants in households that moved in less than 4 years ago	IM_emménage_moins_4ans
Proportion of inhabitants in households that moved in more than 10 years ago	IM_emménage_plus_10ans
Proportion of children aged 2 to 17 enrolled in school among the population of 2 to 17-year-olds	ED_2a17ans_scol
Proportion of students aged 15 to 64 in the population of 15-64-year-olds	ED_15ans_etplus_scol
Proportion of inhabitants without a diploma or holding a certificate among those over 15 years old (not in school)	ED_sansDip
Proportion of inhabitants with a CAP, BEP among those over 15 years old (not in school)	ED_CAP_BEP
Proportion of inhabitants with a baccalaureate, professional certificate among those over 15 years old (not in school)	ED_Bac
Proportion of inhabitants with a higher education diploma (at least a 2-year degree) among those over 15 years old (not in school)	ED_DipSup
Average number of rooms in main residences	LOG_pieces_res
Proportion of main residences built before 1970	LOG_res_av1970
Proportion of main residences built from 1991 (maximum 2017)	LOG_res_apr1990
Proportion of main residences under 40m²	LOG_res_moins40m
Proportion of main residences over 100m²	LOG_res_plus100m
Proportion of main residences without a bathtub or shower	LOG_res_sans_douche
Proportion of main residences without central heating or individual electric heating	LOG_res_sans_chauffage
Proportion of households with a parking space	LOG_men_stationnement
Proportion of households without a car	LOG_men_sans_voiture
Proportion of households with two or more cars	LOG_men_voitures_2plus
Proportion of main residences (excluding studios) with 1 person in overcrowding	LOG_res_surrocup
Proportion of secondary residences and occasional housing among the total housing stock	LOG_res_secondaires
Proportion of people living in empty low-rent housing main residences among the total population	LOG_habitants_hlm_loué
Average number of rooms in main residences	LOG_nombre_pieces_personnes
Proportion of houses in housing stock	LOG_maisons
Proportion of apartments in housing stock	LOG_apparts
Proportion of people who don't own their main residence	LOG_res_non_proprio
Proportion of active men in the male population	ER_hommes_actifs
Proportion of active women in the female population	ER_femmes_actives
Proportion of active inhabitants in the total population	ER_pop_actifs
Proportion of unemployed inhabitants aged 15-24 among the active population of 15-24 years old	ER_chom_jeunes
Proportion of unemployed inhabitants aged 55-64 among the population of 55-64 years old	ER_chom_agé
Proportion of unemployed inhabitants in the active population	ER_chom
Proportion of farmers in the population aged 15 and older	ER_agric
Proportion of craftsmen in the population aged 15 and older	ER_artisan
Proportion of white-collar workers in the population aged 15 and older	ER_cadres
Proportion of intermediate professions in the population aged 15 and older	ER_profession_inter
Proportion of employees in the population aged 15 and older	ER_employe
Proportion of blue-collar workers in the population aged 15 and older	ER_ouvriers
Proportion of retirees in the population aged 15 and older	ER_retraites
Proportion of inhabitants without professional activity in the population aged 15 and older	ER_sans_activite_pro
Proportion of part-time workers in the population aged 15 and older	ER_temps_partiel
Proportion of self-employed inhabitants in the population aged 15 and older	ER_non_salarie
Proportion of inhabitants on permanent contracts (or in the public sector) in the active population	ER_cdi
Proportion of unstable employment in the active population (internships, fixed-term contracts, aid jobs, temporary work, apprenticeships)	ER_emploi_instable
Median income	ER_revenu_median
European deprivation index	EDI

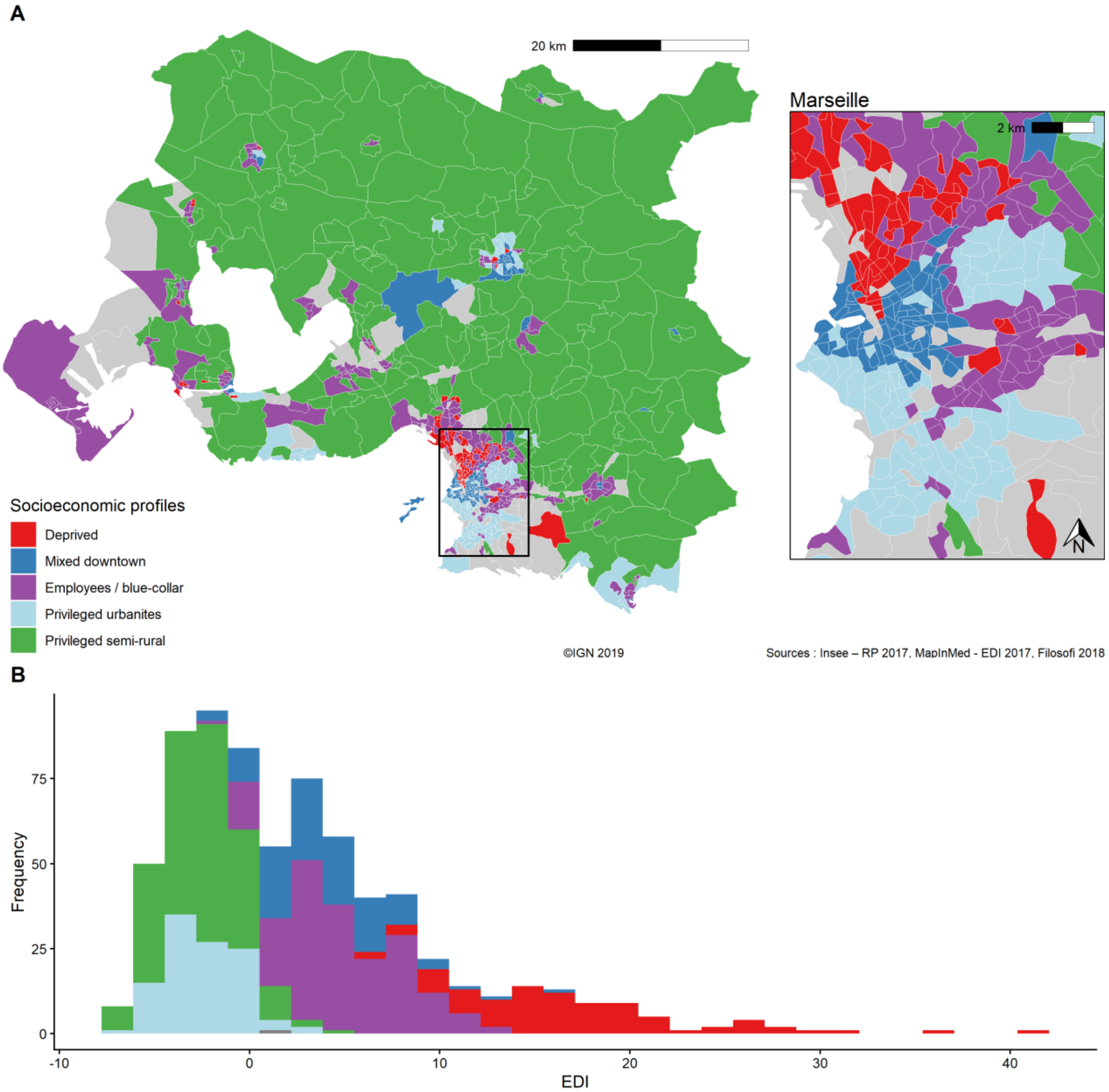

Fig 2: (A) Spatial distribution of the 5 socioeconomic profiles in the Aix–Marseille area (B) and histogram of the European Deprivation Index (EDI), with the histogram coloured according to the profiles. Deprived (n=89), mixed downtown (n=109), employees / blue collar (n=190), privileged urbanites (n=108), privileged semi-rural (n=208)

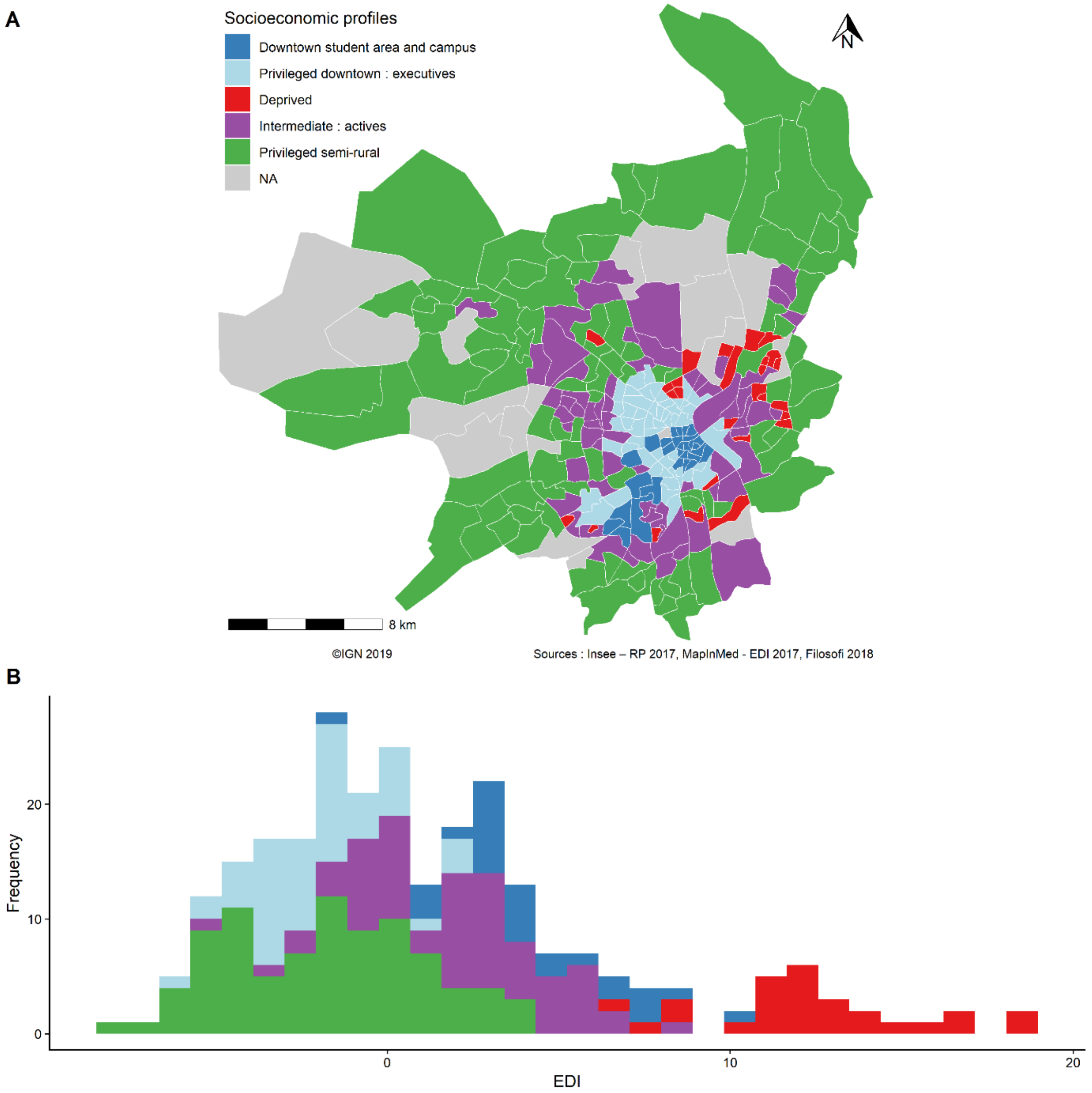

Fig 3: (A) Spatial distribution of the 5 socioeconomic profiles of Bordeaux (B) and histogram of the European Deprivation Index (EDI), with the histogram coloured according to the profiles. Privileged semi-rural (n=87), privileged downtown : executives (n=52), intermediate : actives (n=65), deprived (n=27), mi downtown (n=109), downtown student area and campus (n=28)

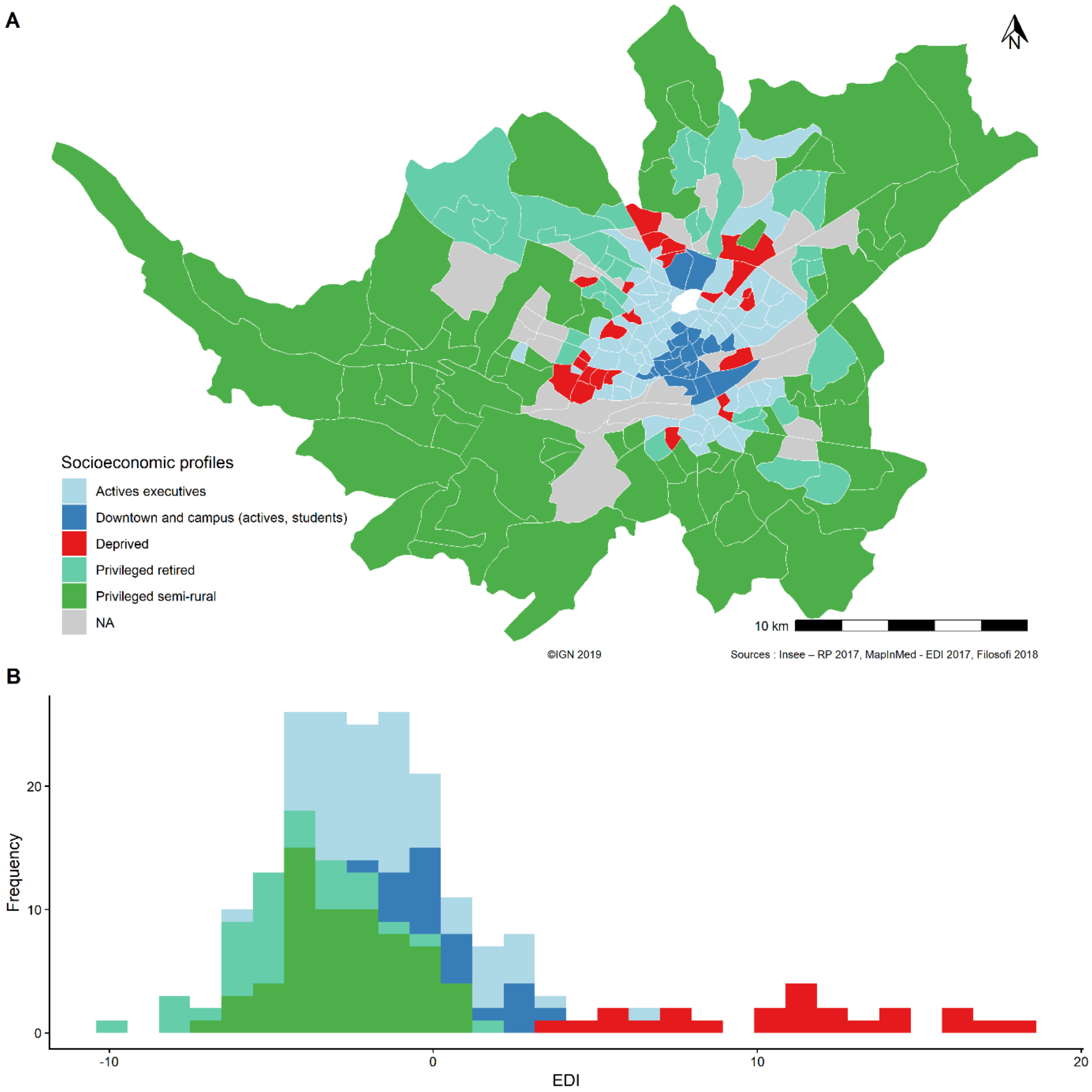

Fig 3: (A) Spatial distribution of the 5 socioeconomic profiles of Nantes (B) and histogram of the European Deprivation Index (EDI), with the histogram coloured according to the profiles. privileged retired (n=33), privileged semi-rural : (n=62), intermediate : actives (n=65), actives executives (n=65), downtown and campus (actives, students) (n=22), deprived (n=23)

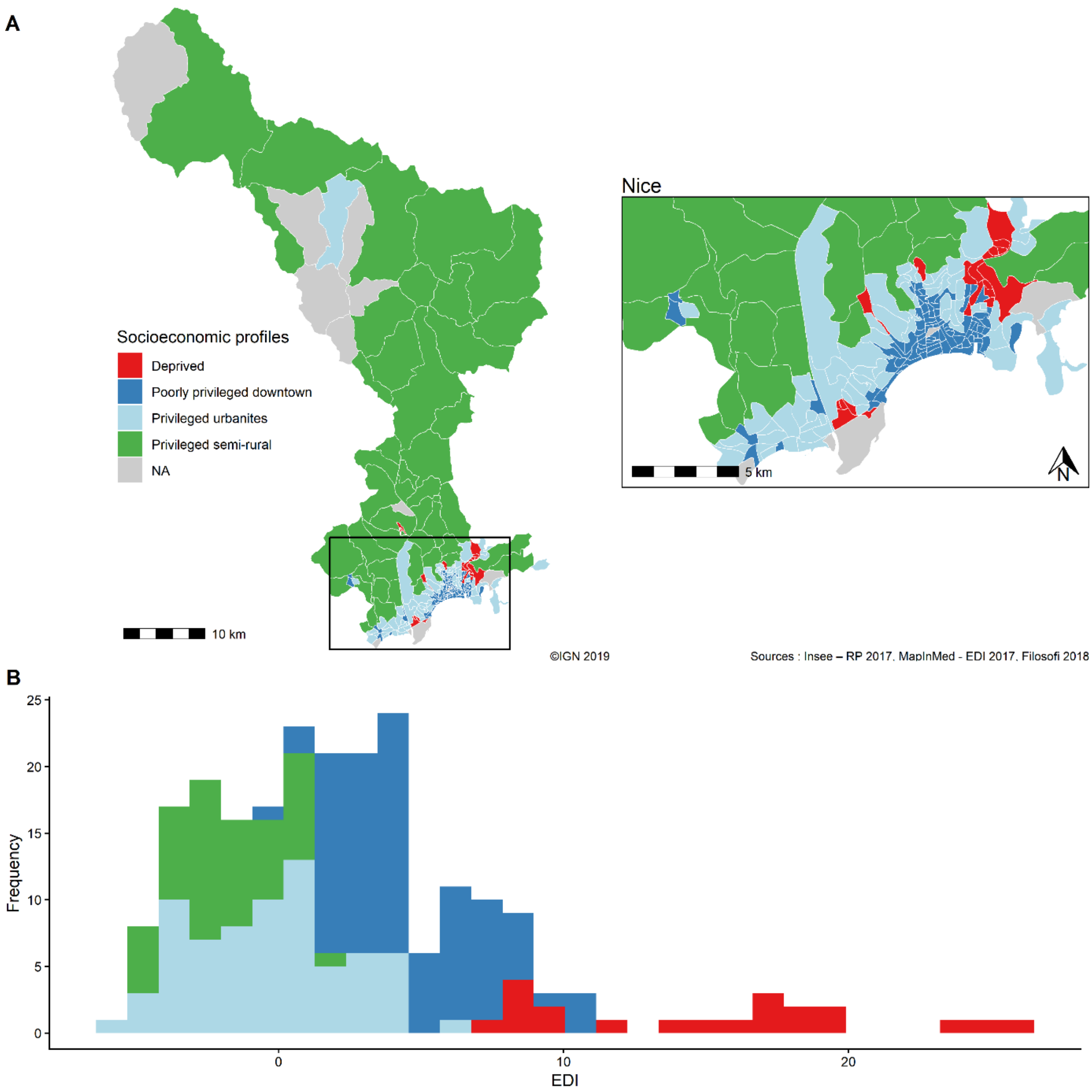

Fig 4: (A) Spatial distribution of the 4 socioeconomic profiles of Nice (B) and histogram of the European Deprivation Index (EDI), with the histogram coloured according to the profiles. Deprived (n=22), poorly privileged downtown (n=85), privileged urbanites (n=70), privileged semi-rural (n=47)

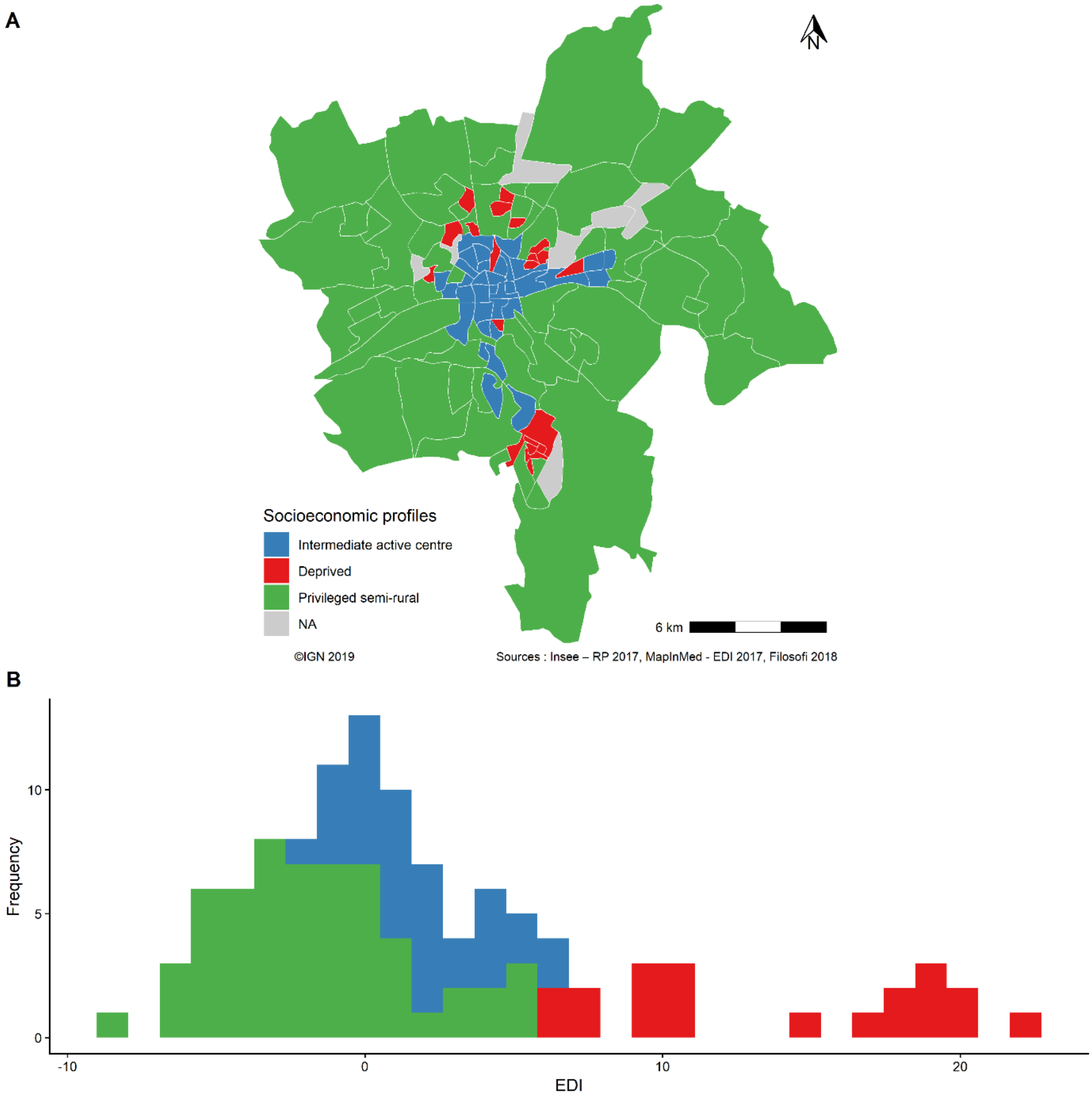

Fig 5: (A) Spatial distribution of the 3 socioeconomic profiles of Orleans (B) and histogram of the European Deprivation Index (EDI), with the histogram coloured according to the profiles. Deprived (n=20), intermediate active centre (n=33), privileged semi-rural (n=57)

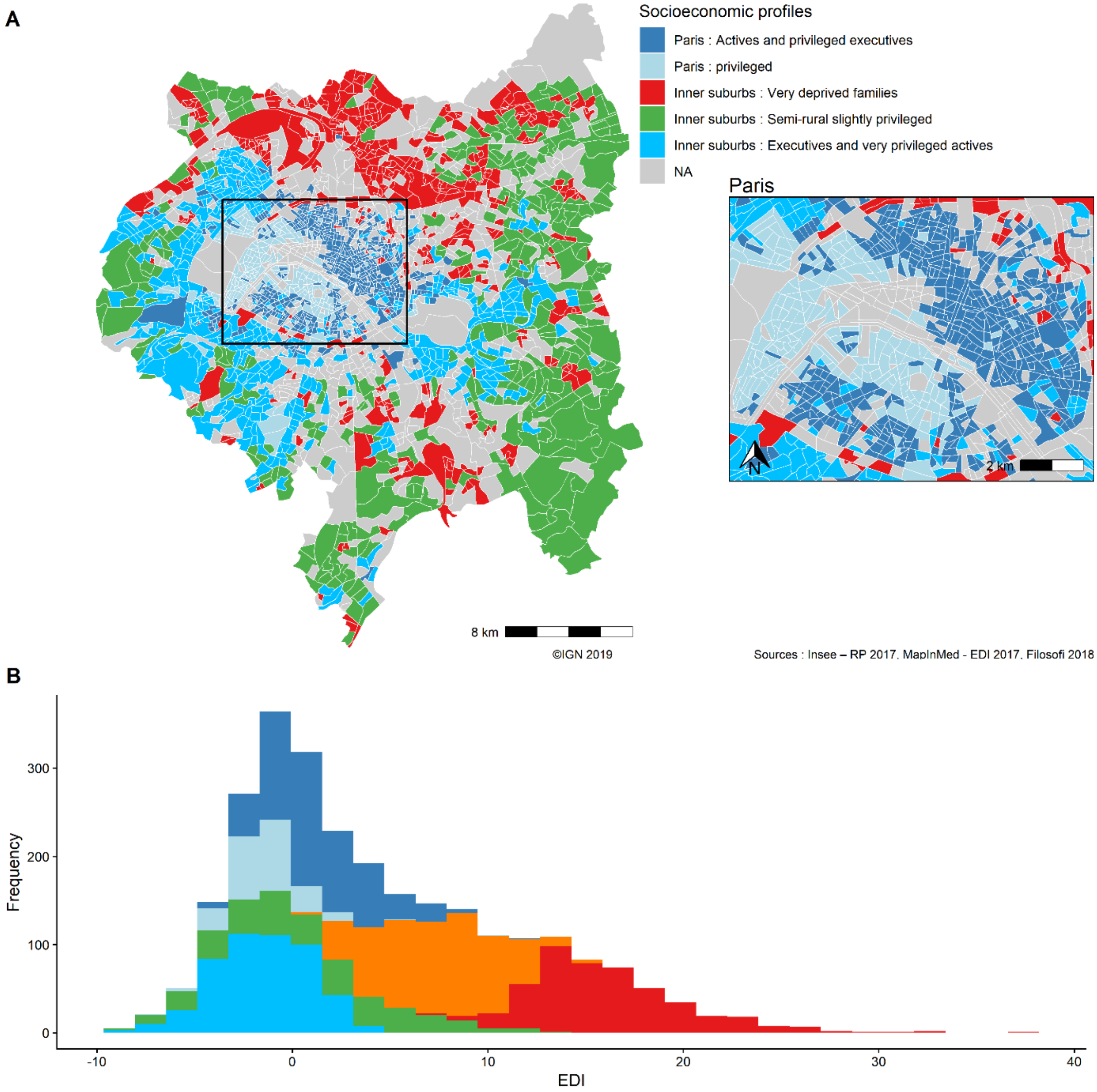

Fig 6: (A) Spatial distribution of the 6 socioeconomic profiles of Grand Paris (B) and histogram of the European Deprivation Index (EDI), with the histogram coloured according to the profiles. Paris : privileged (n=223), Paris : actives and privileged executives (n=548), Inner suburbs : executives and very privileged actives (n=497), Inner suburbs : semi-rural slightly privileged (n=334), Inner suburbs : Deprived actives (n=601), Inner suburbs : very deprived families (n=469)

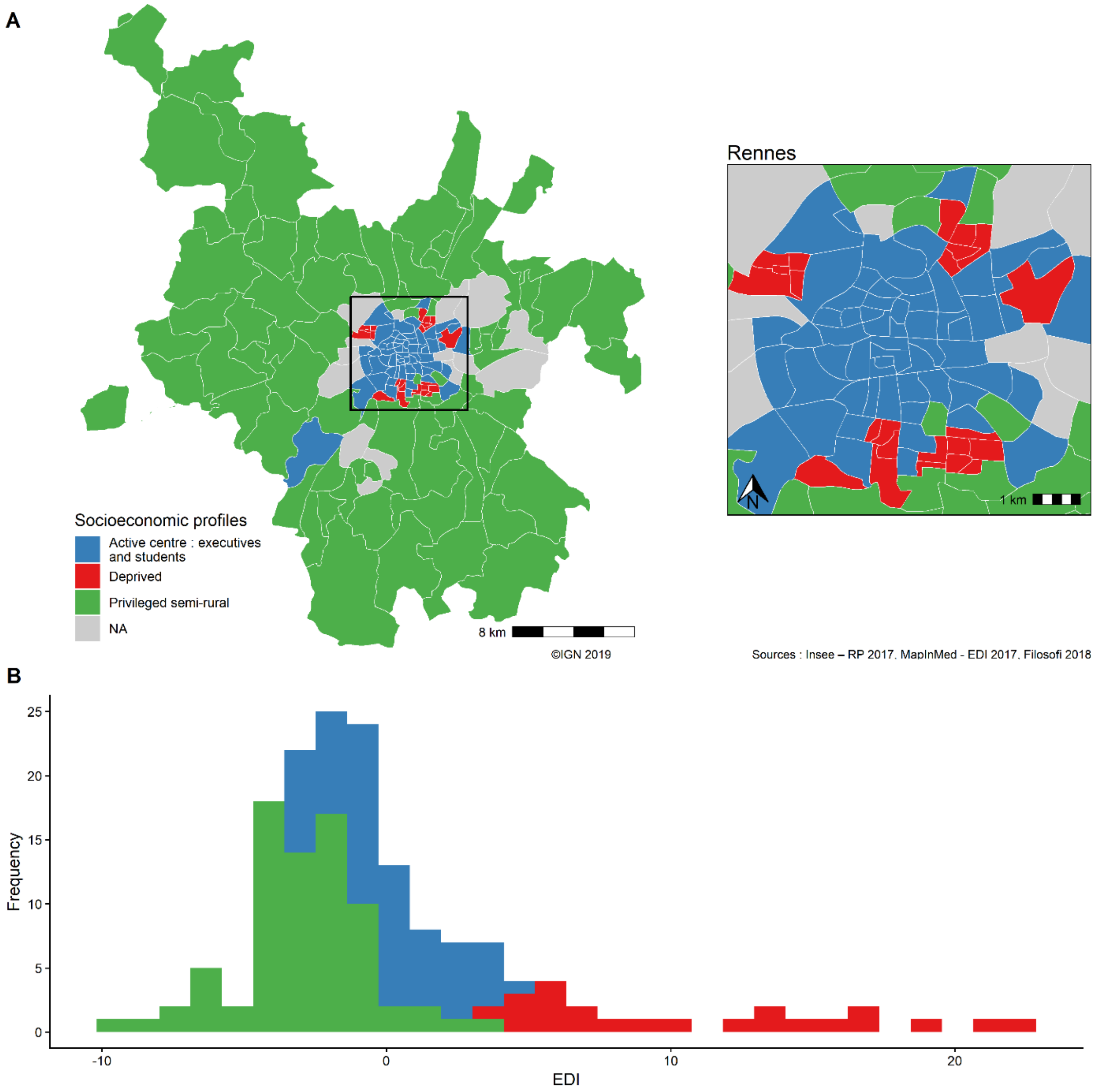

Fig 7: (A) Spatial distribution of the 3 socioeconomic profiles of Rennes (B) and histogram of the European Deprivation Index (EDI), with the histogram coloured according to the profiles. deprived (n=23), active centre : executives and students (n=59), privileged semi-rural (n=76)

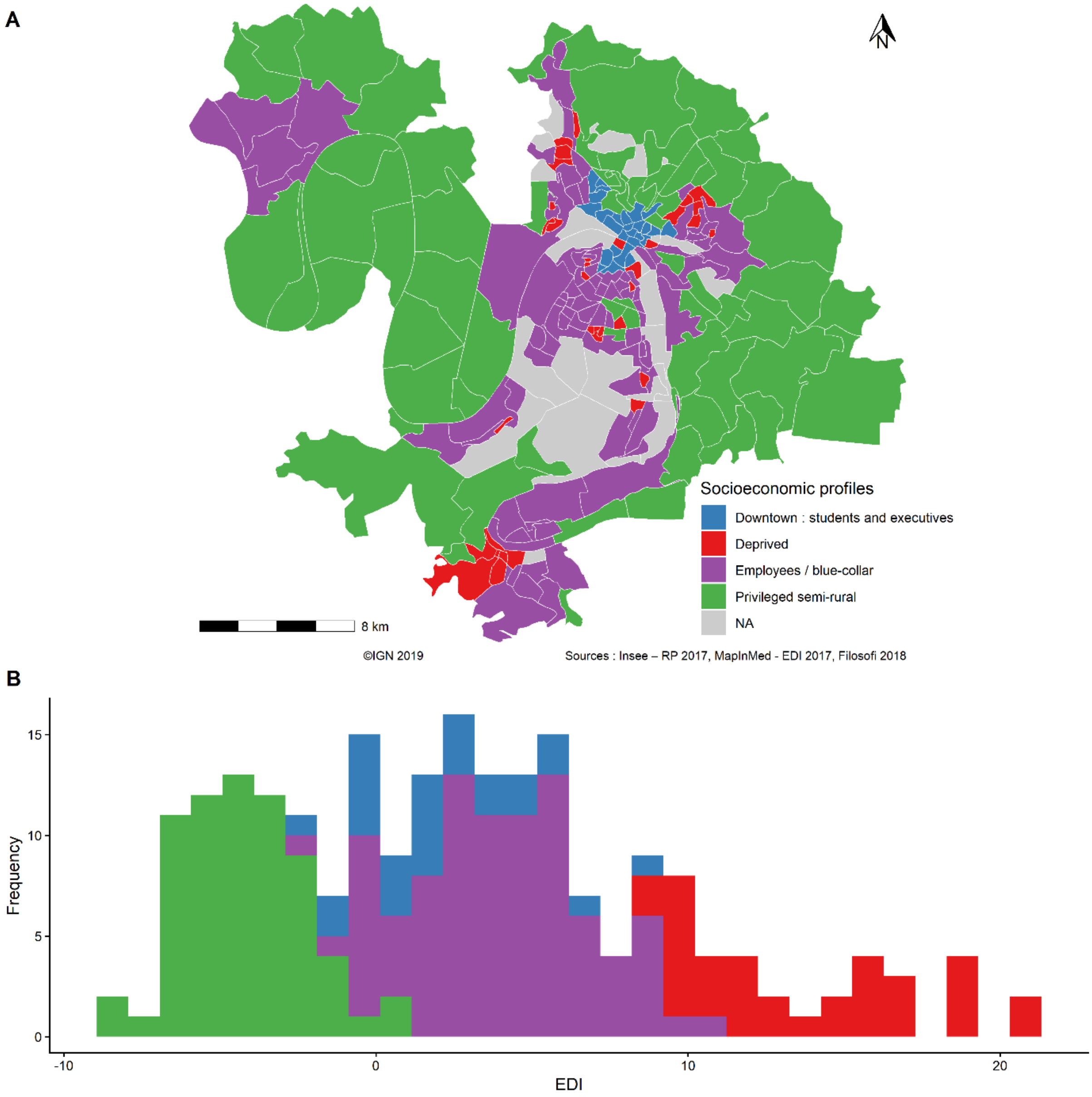

Fig 8: (A) Spatial distribution of the 4 socioeconomic profiles of Rouen and (B) histogram of the European Deprivation Index (EDI), with the histogram coloured according to the profiles. deprived (n=34), downtown : students and executives (n=27), employees / blue-collar (n=89) , privileged semi-rural (n=67)

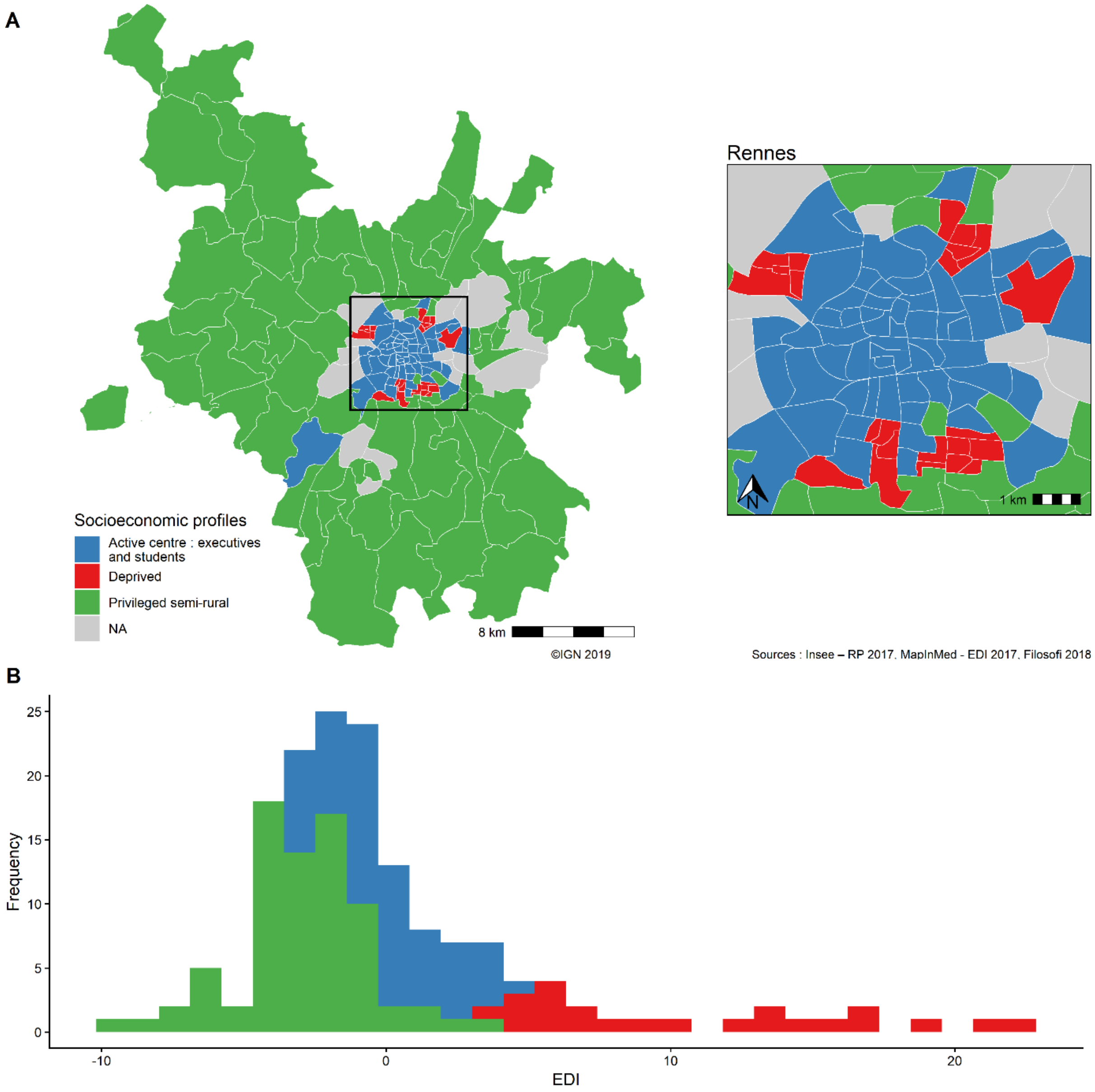

Fig 9: (A) Spatial distribution of the 4 socioeconomic profiles of Rouen and (B) histogram of the European Deprivation Index (EDI), with the histogram coloured according to the profiles. deprived (n=34), downtown: students and executives (n=27), employees / blue-collar (n=89) , privileged semi-rural (n=67)

A

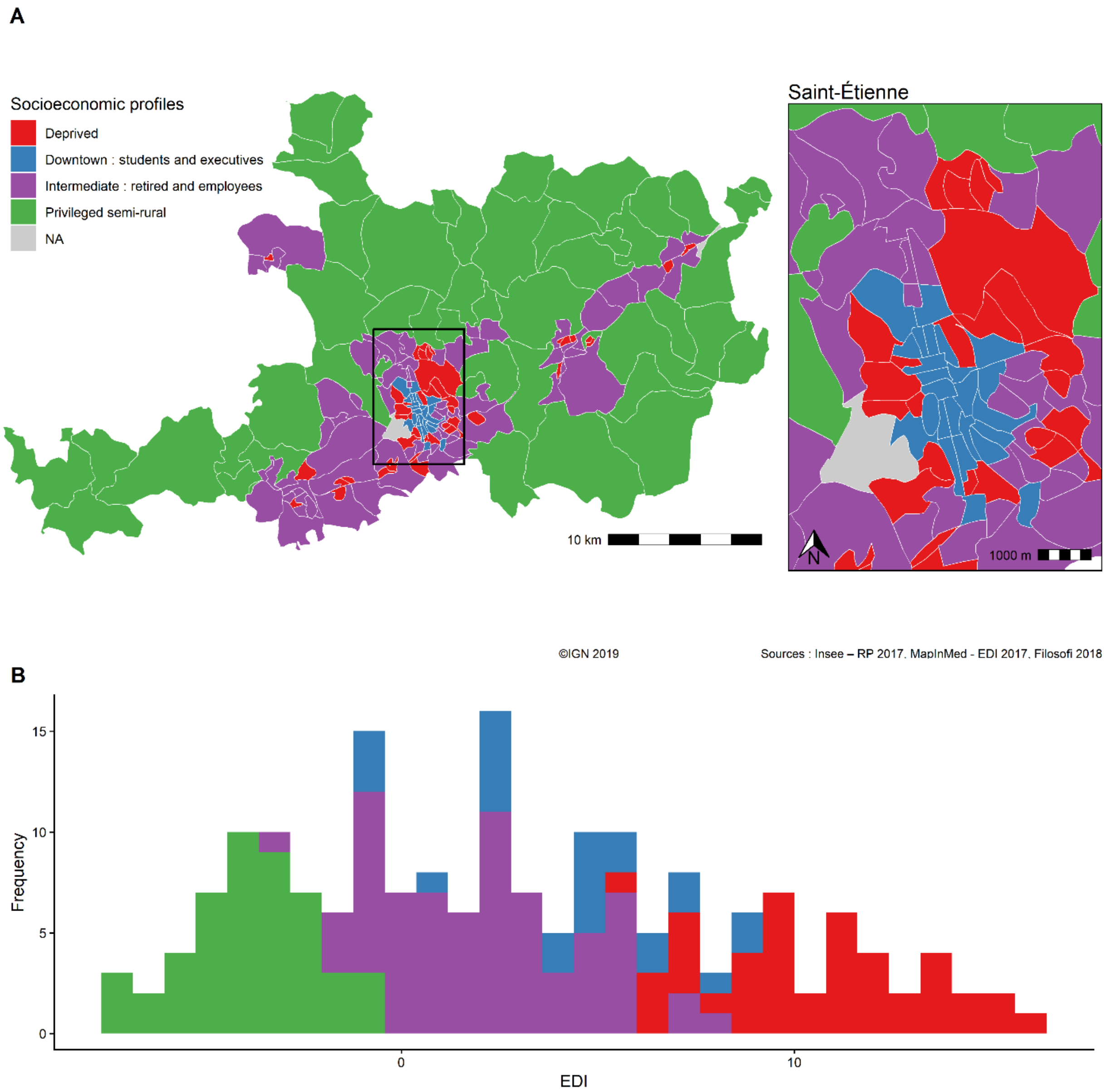

Fig 10: (A) Spatial distribution of the 4 socioeconomic profiles of Saint-Etienne and (B) histogram of the European Deprivation Index (EDI), with the histogram coloured according to the profiles. deprived (n=43), downtown: students and executives (n=25), intermediate: retired and employees (n=69), privileged semi-rural (n=57)

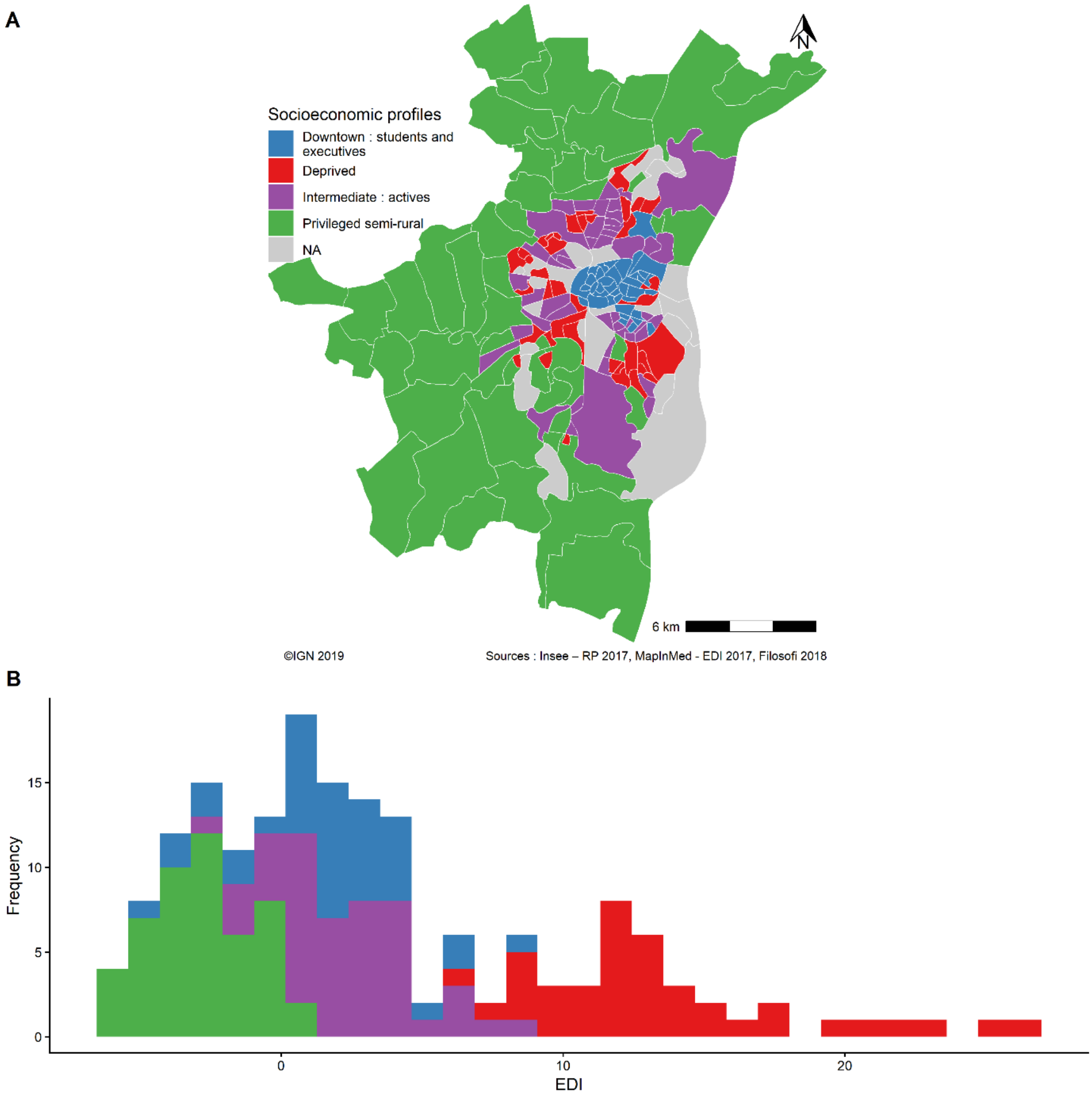

Fig 11: (A) Spatial distribution of the 4 socioeconomic profiles of Strasbourg and (B) histogram of the European Deprivation Index (EDI), with the histogram coloured according to the profiles. deprived (n=40), downtown : students and executives (n=38), intermediate : actives (n=47), privileged semi-rural (n=49)

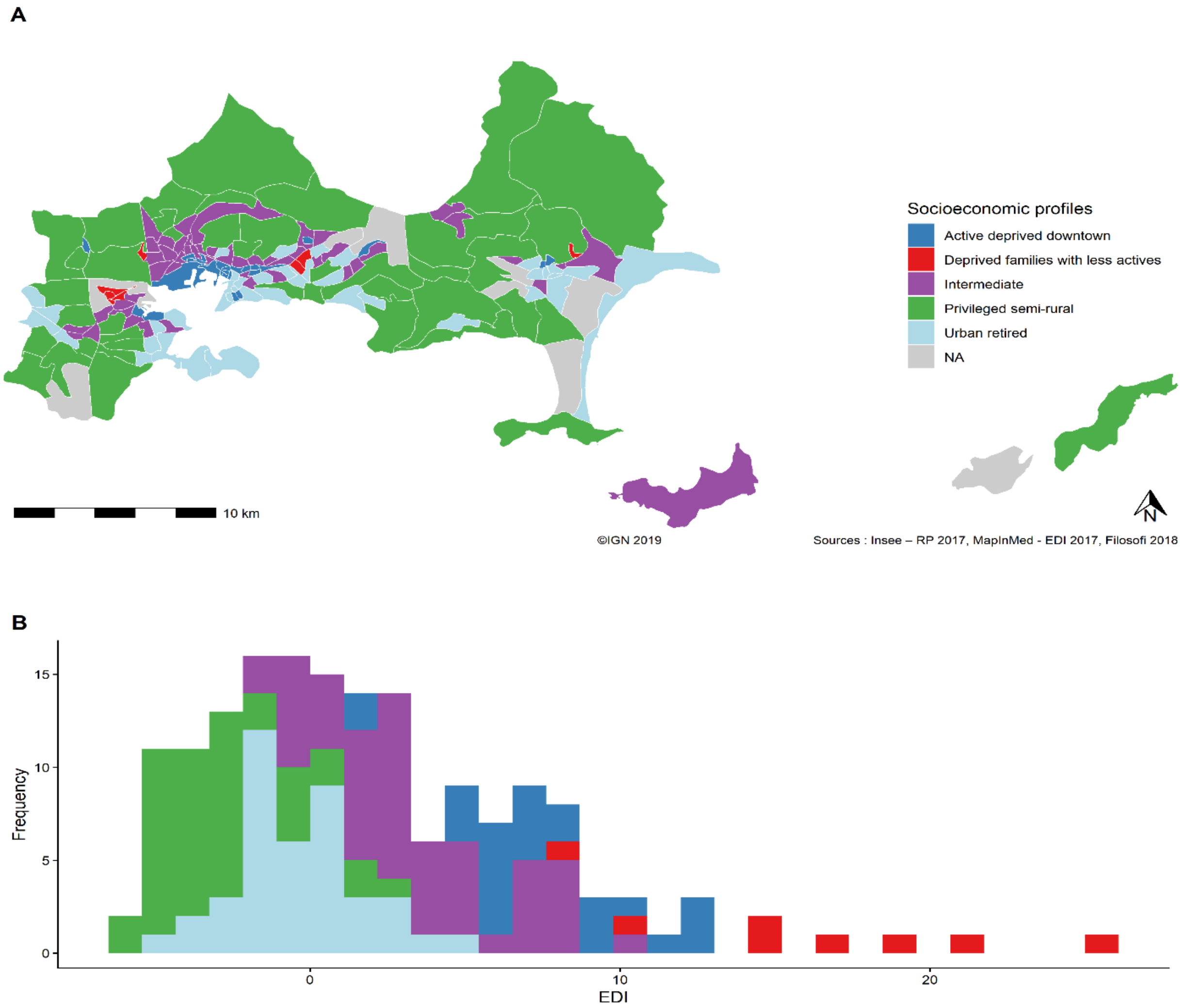

Fig 12: (A) Spatial distribution of the 5 socioeconomic profiles of Toulon and (B) histogram of the European Deprivation Index (EDI), with the histogram coloured according to the profiles. privileged semi-rural (n=42), urban retired (n=41), intermediate (n=51), active deprived downtown (n=25) , deprived families with less activities (n=8)

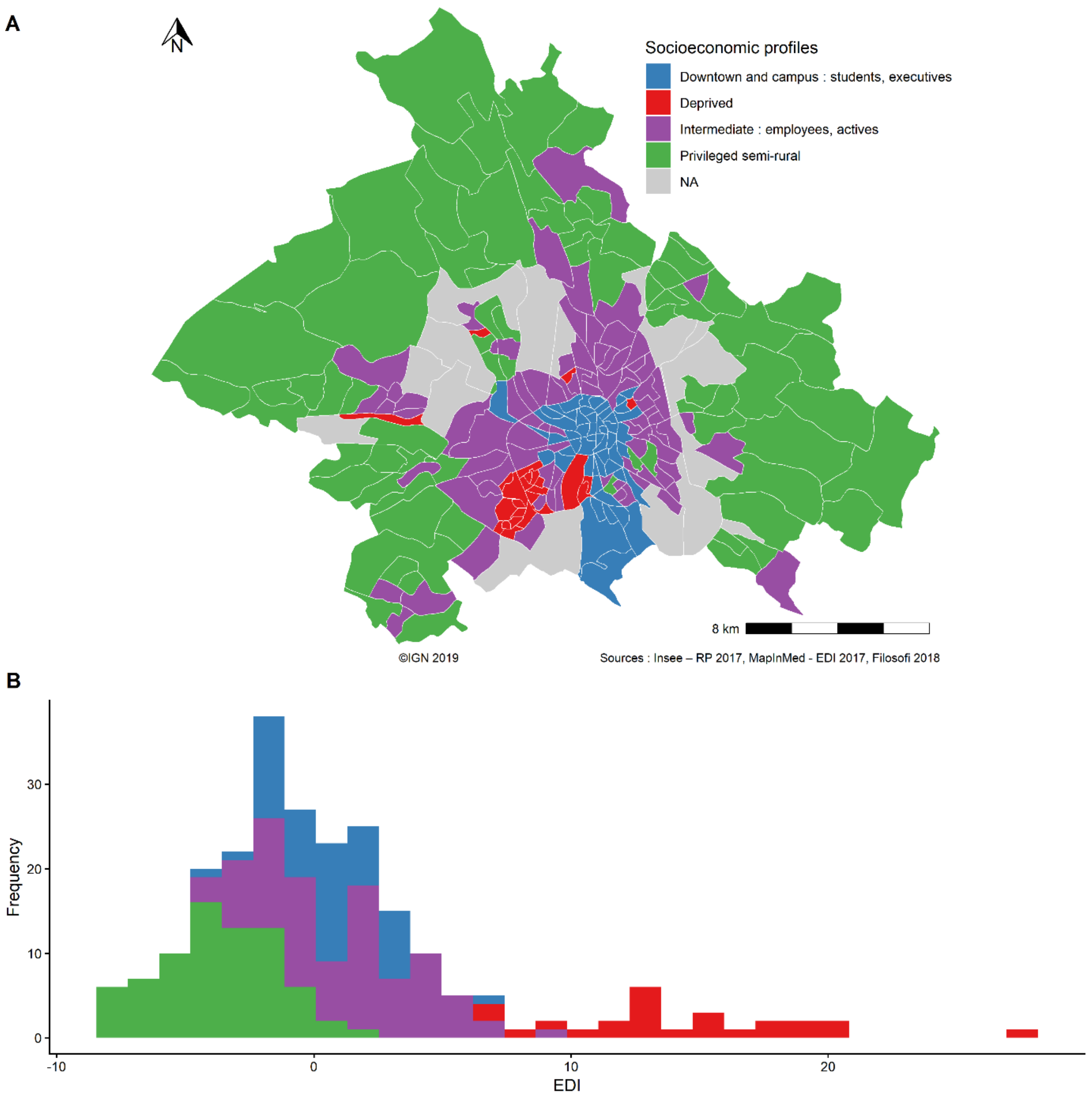

Fig 13: (A) Spatial distribution of the 4 socioeconomic profiles of Toulouse and (B) histogram of the European Deprivation Index (EDI), with the histogram coloured according to the profiles. privileged semi-rural (n=74), intermediate: employees, actives (n=86), downtown and campus: students, executives (n=52) , deprived (n=25)

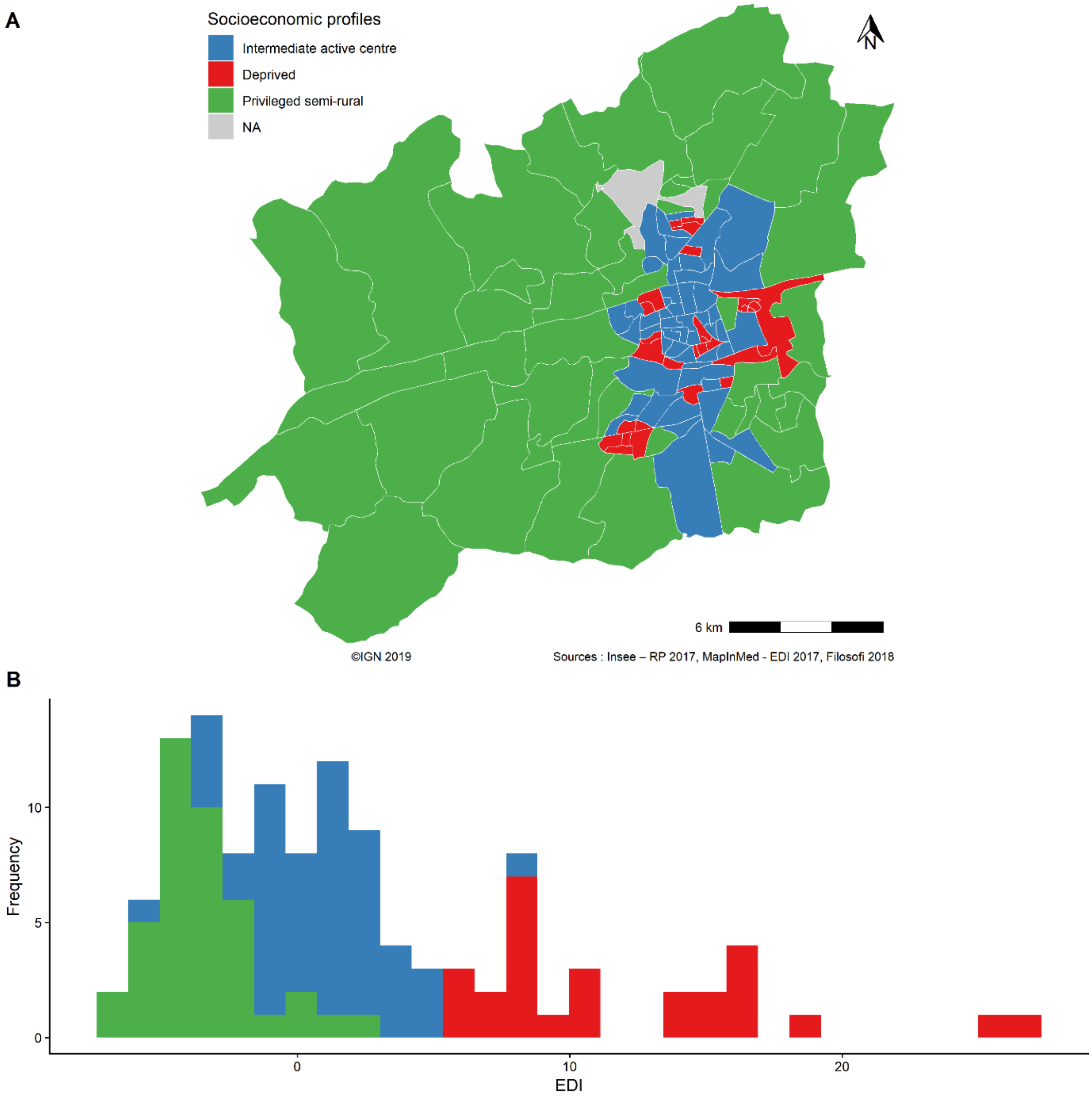

Fig 14: (A) Spatial distribution of the 3 socioeconomic profiles of Tours and (B) histogram of the European Deprivation Index (EDI), with the histogram coloured according to the profiles. privileged semi-rural (n=50), intermediate active centre (n=41), deprived (n=27)

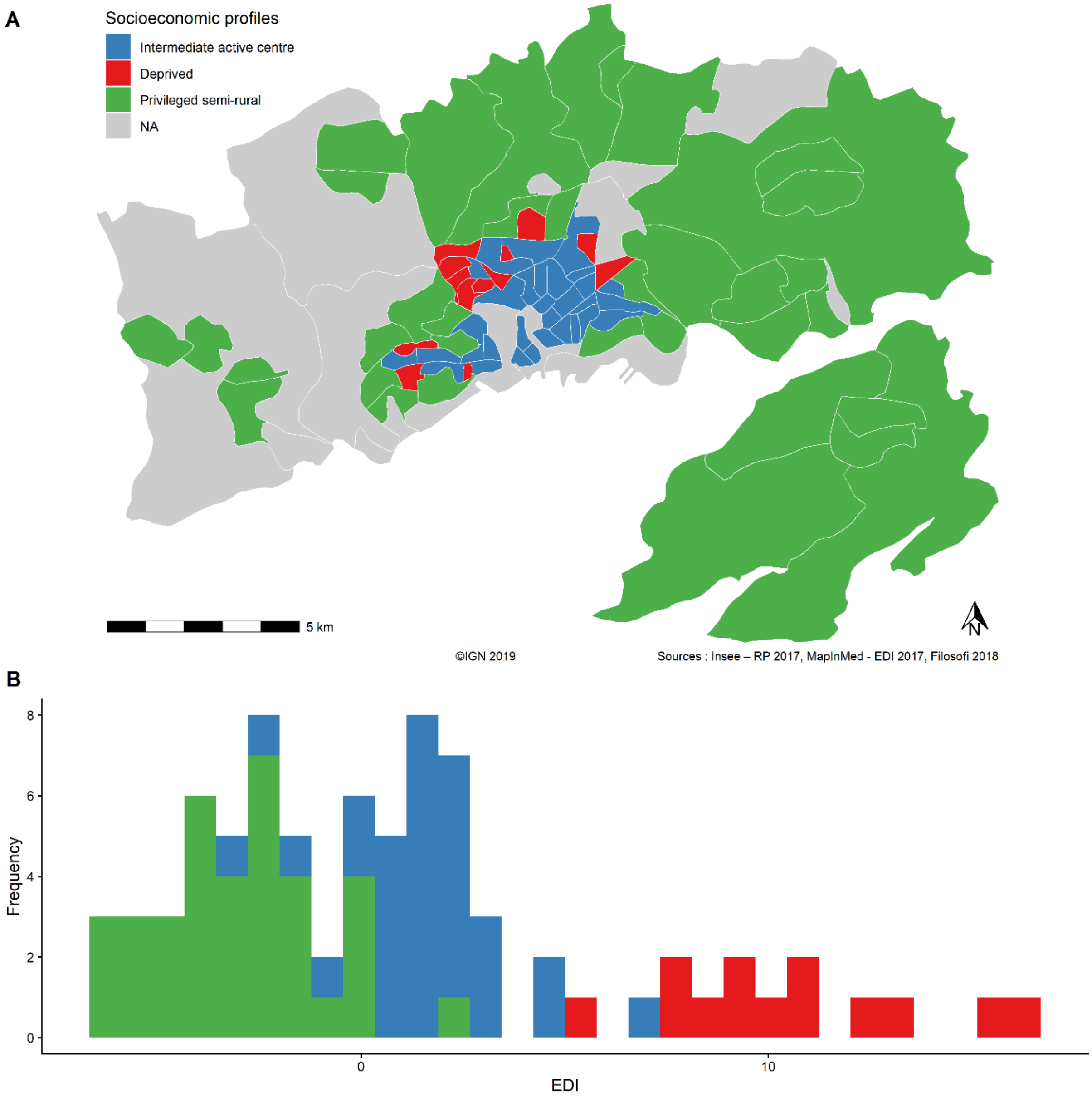

Fig 15: (A) Spatial distribution of the 3 socioeconomic profiles of Brest and (B) histogram of the European Deprivation Index (EDI), with the histogram coloured according to the profiles. deprived (n=13), intermediate active centre (n=31), privileged semi-rural (n=36)

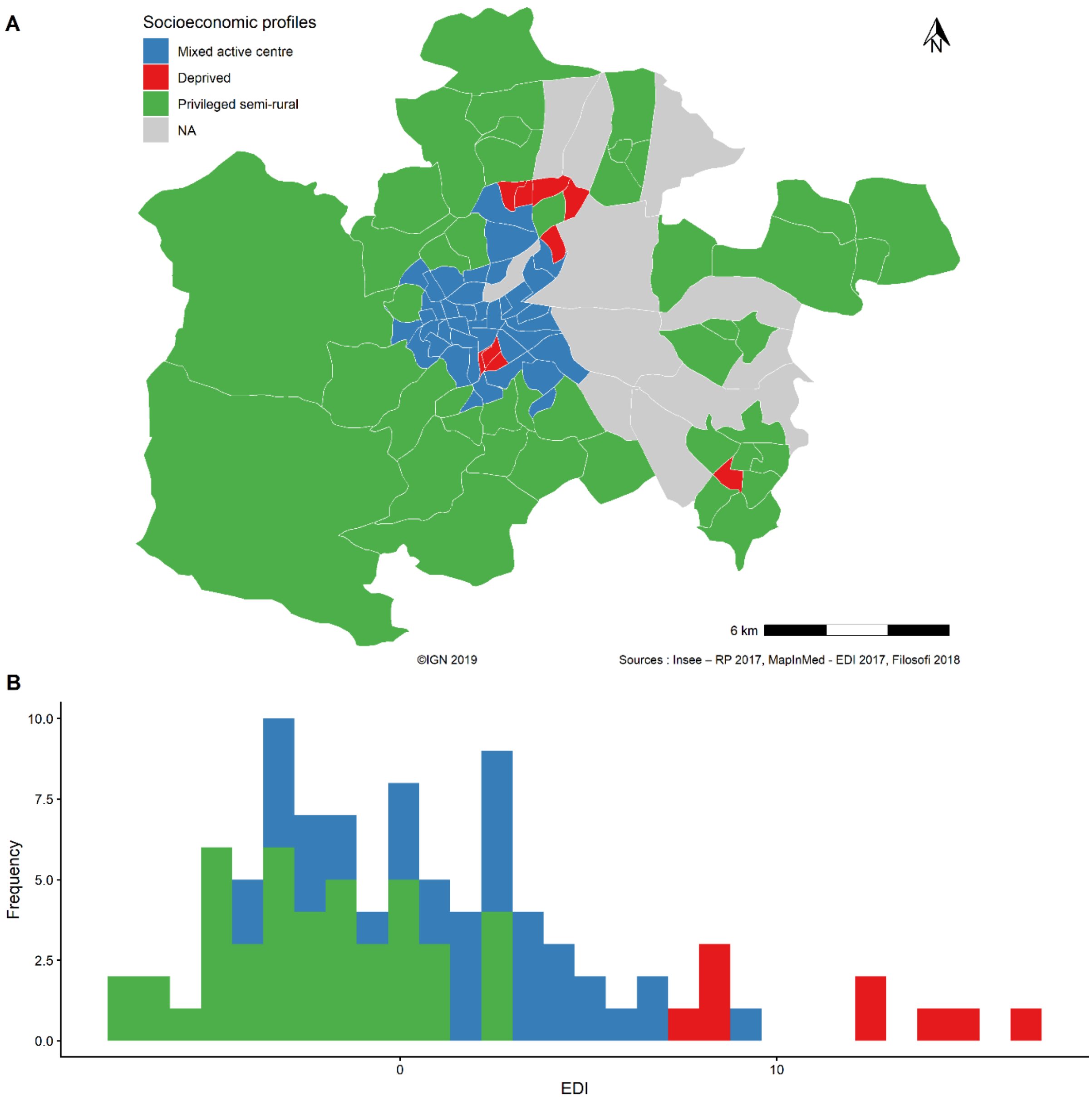

Fig 16: (A) Spatial distribution of the 3 socioeconomic profiles of Clermont Auvergne and (B) histogram of the European Deprivation Index (EDI), with the histogram coloured according to the profiles. privileged semi-rural (n=44), mixed active centre (n=39), deprived (n=9)

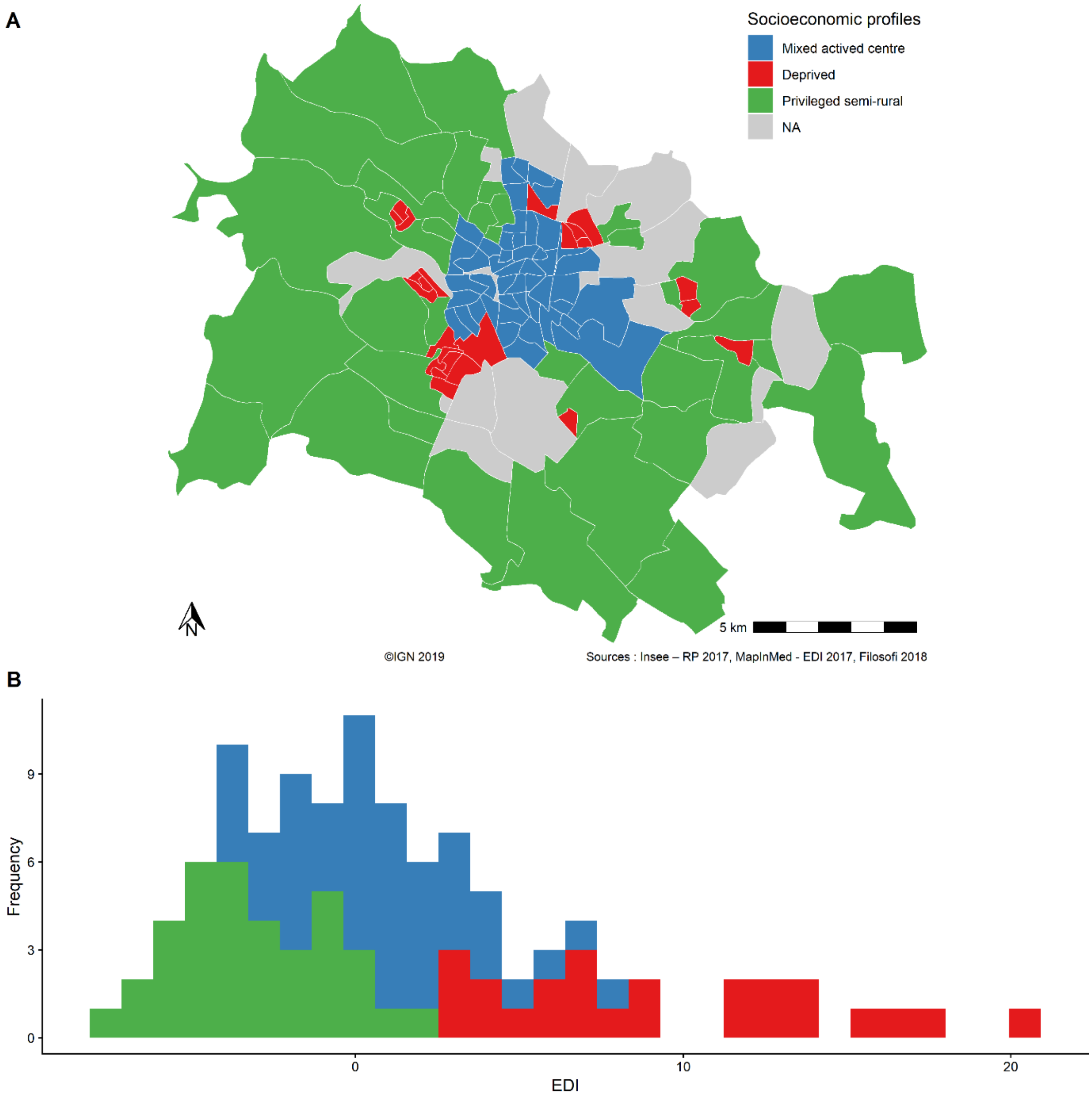

Fig 17: (A) Spatial distribution of the 3 socioeconomic profiles of Dijon and (B) histogram of the European Deprivation Index (EDI), with the histogram coloured according to the profiles. privileged semi-rural (n=36), mixed active centre (n=47), deprived (n=24)

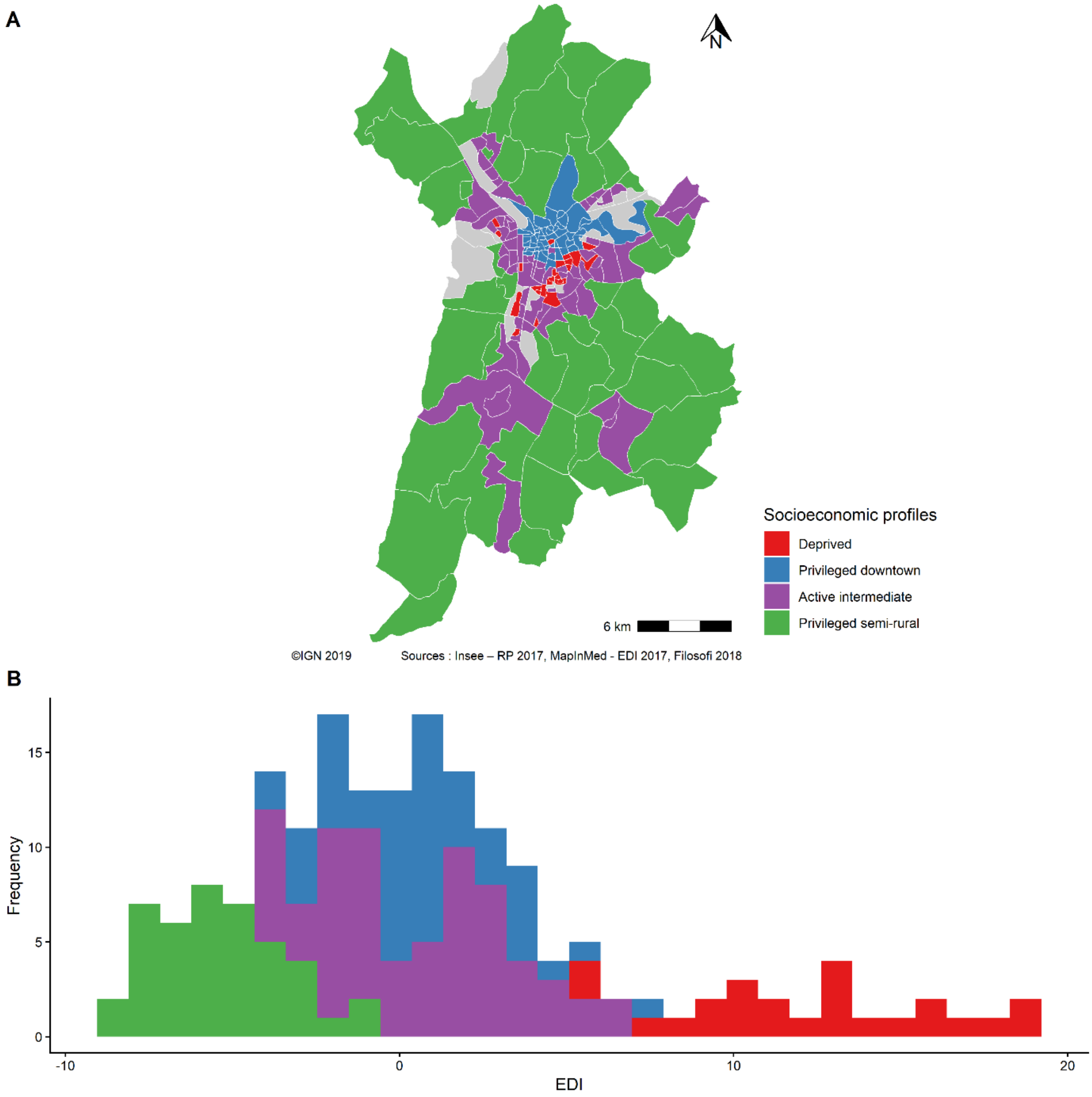

Fig 18: (A) Spatial distribution of the 4 socioeconomic profiles of Grenoble and (B) histogram of the European Deprivation Index (EDI), with the histogram coloured according to the profiles. deprived (n=24), privileged downtown (n=50), active intermediate (n=67), privileged semi-rural (n=42)

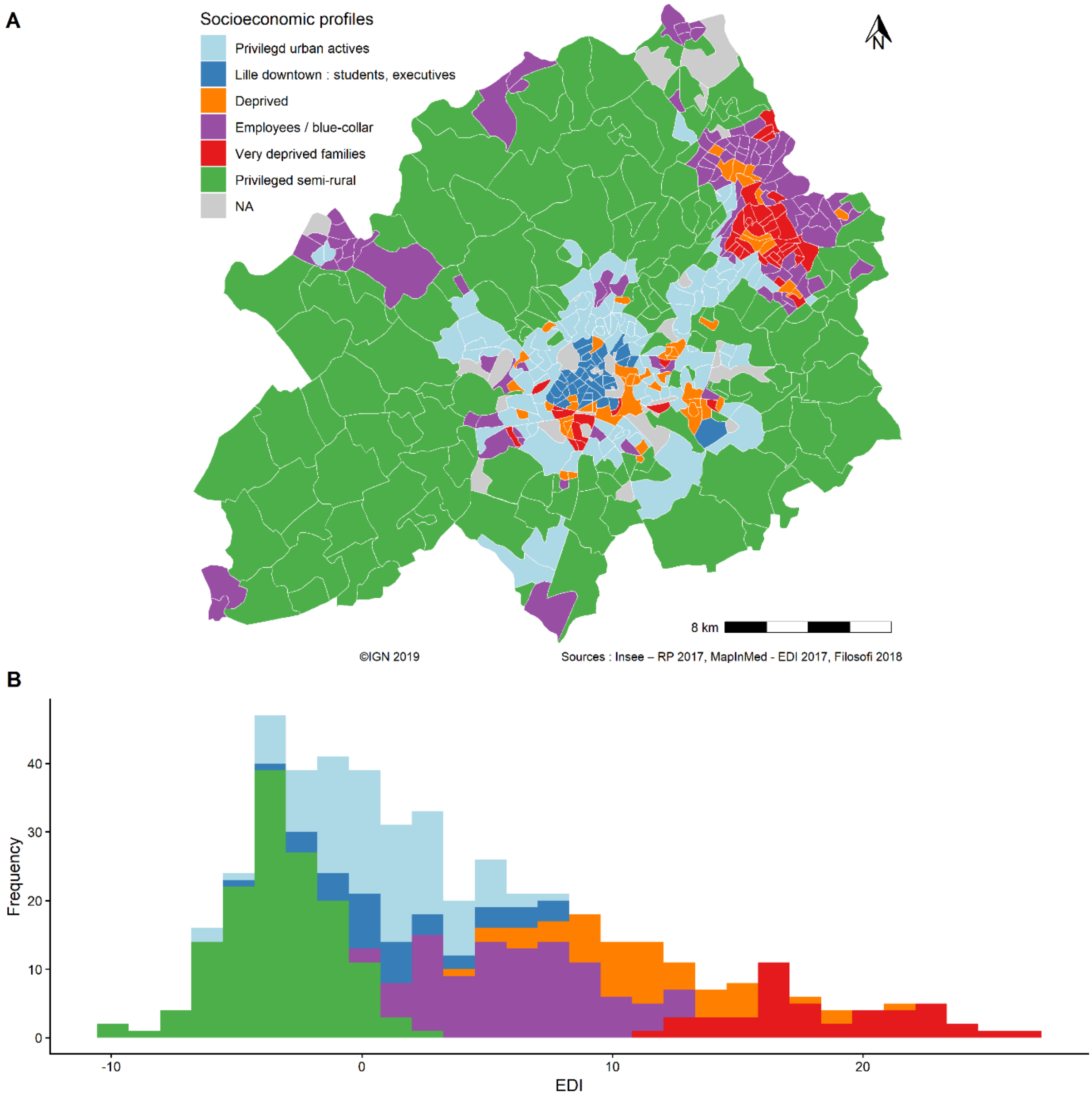

Fig 19: (A) Spatial distribution of the 6 socioeconomic profiles of Lille and (B) histogram of the European Deprivation Index (EDI), with the histogram coloured according to the profiles. privileged semi-rural (n=144), privileged urban actives (n=104), employees / blue-collar (n=96), Lille downtown : students, executives (n=37), deprived (n=50), very deprived families (n=45)

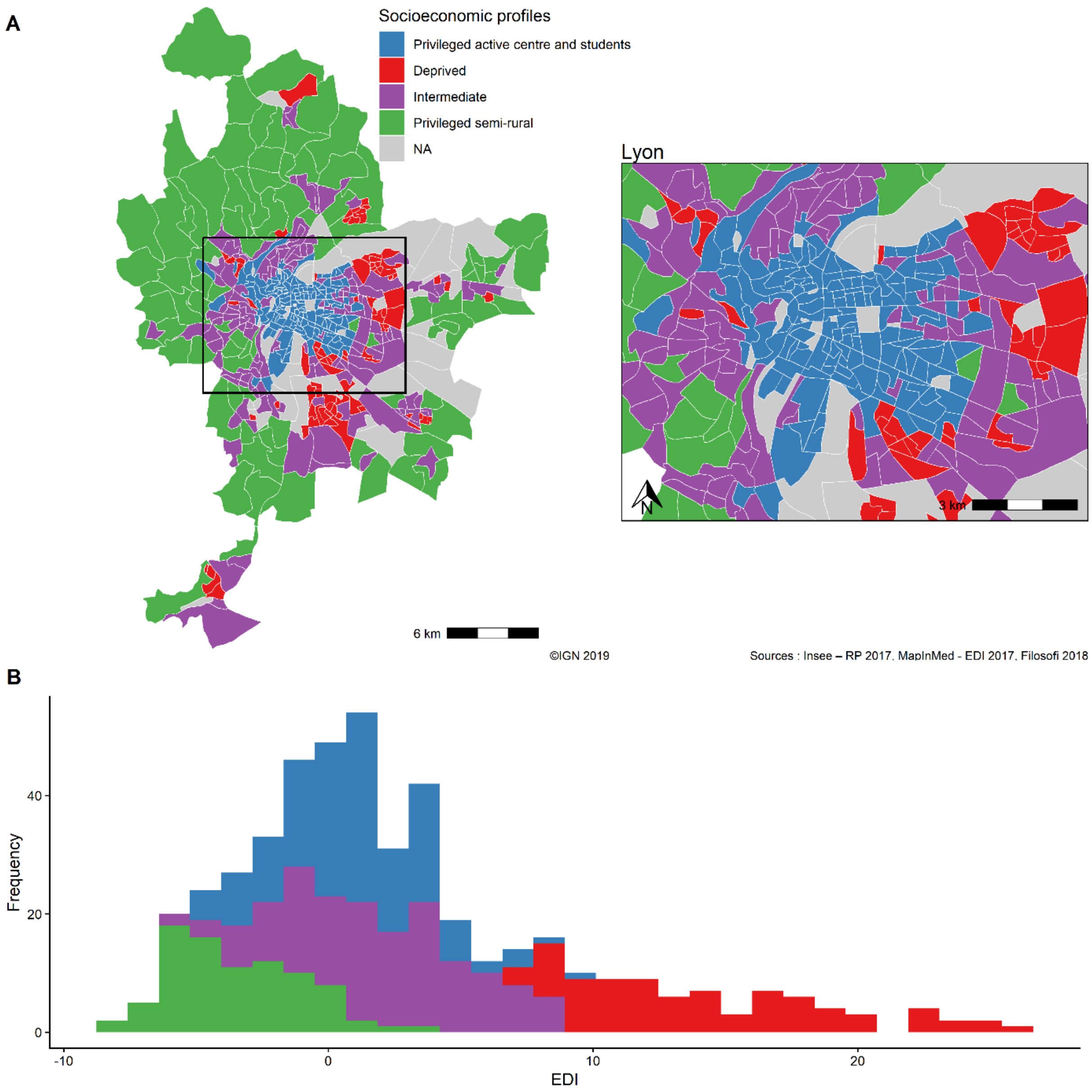

Fig 20: (A) Spatial distribution of the 4 socioeconomic profiles of Lyon and (B) histogram of the European Deprivation Index (EDI), with the histogram coloured according to the profiles. privileged semi-rural (n=86), privileged active centre and students (n=149), intermediate (n=148), deprived (n=84)

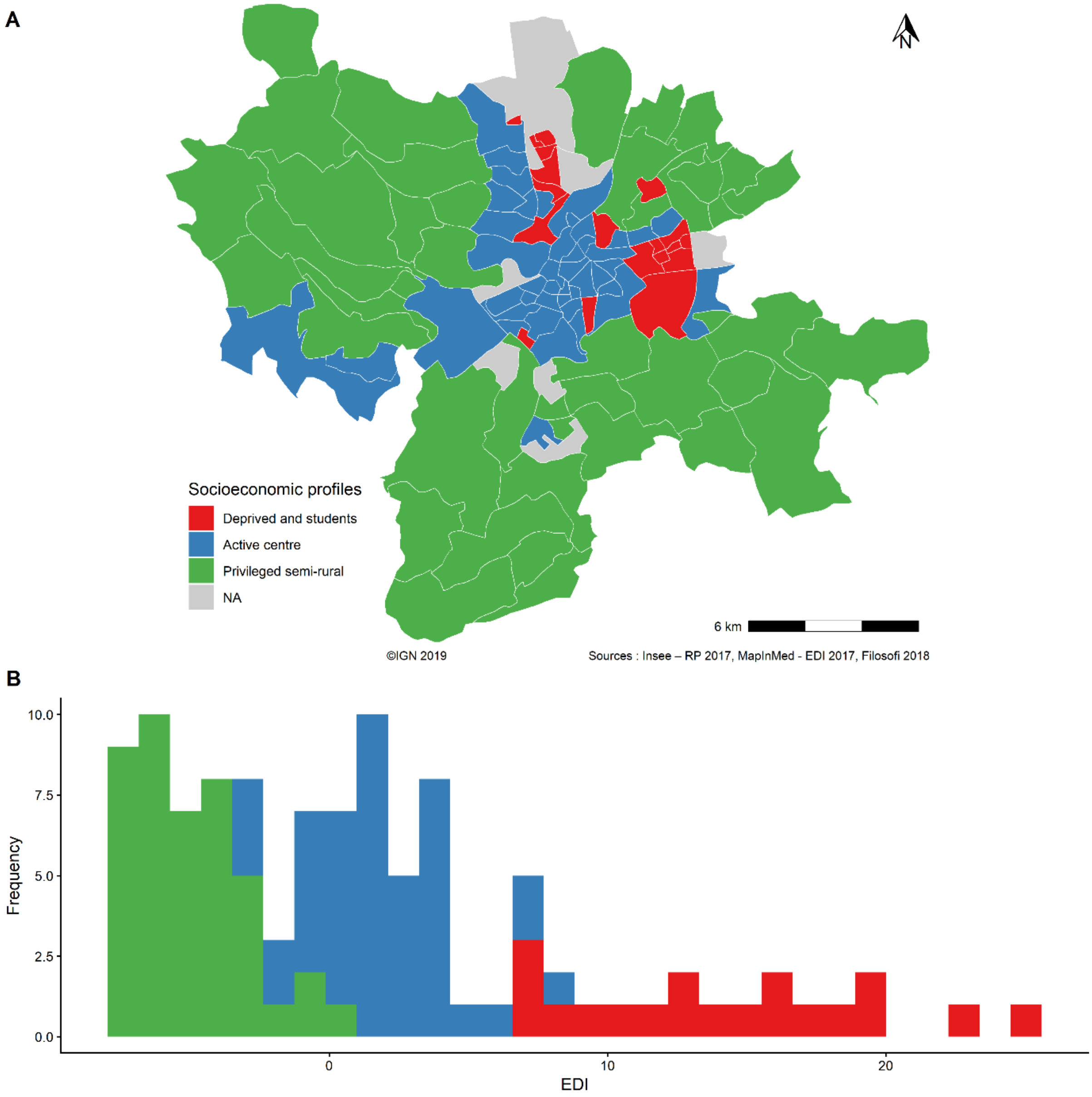

Fig 21: (A) Spatial distribution of the 3 socioeconomic profiles of Metz and (B) histogram of the European Deprivation Index (EDI), with the histogram coloured according to the profiles. deprived and students (n=19), active centre (n=44), privileged semi-rural (n=43)

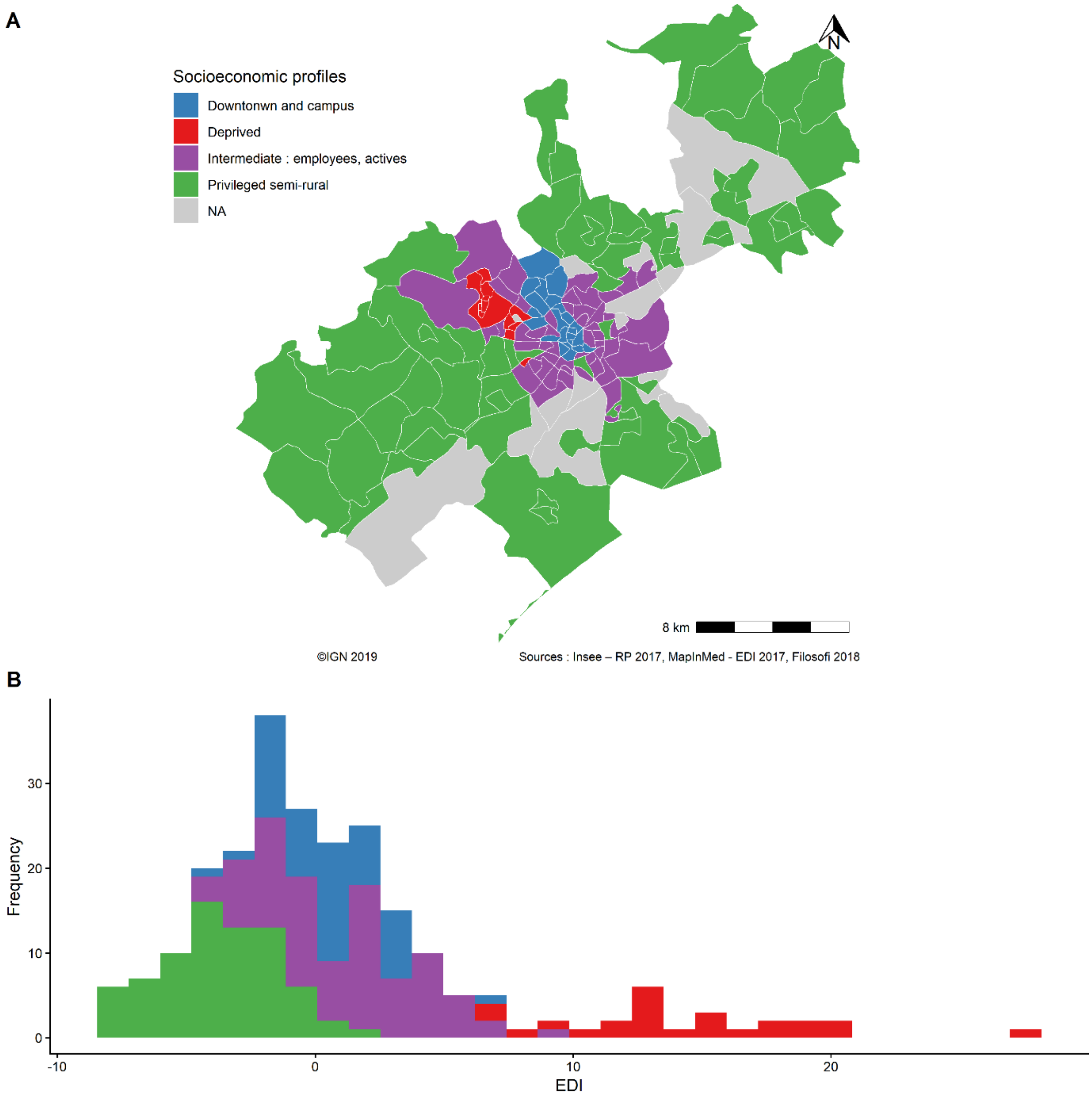

Fig 22: (A) Spatial distribution of the 4 socioeconomic profiles of Montpellier and (B) histogram of the European Deprivation Index (EDI), with the histogram coloured according to the profiles. deprived (n=13), downtown and campus (n=22), intermediate : employees, actives (n=49), privileged semi-rural (n=60)

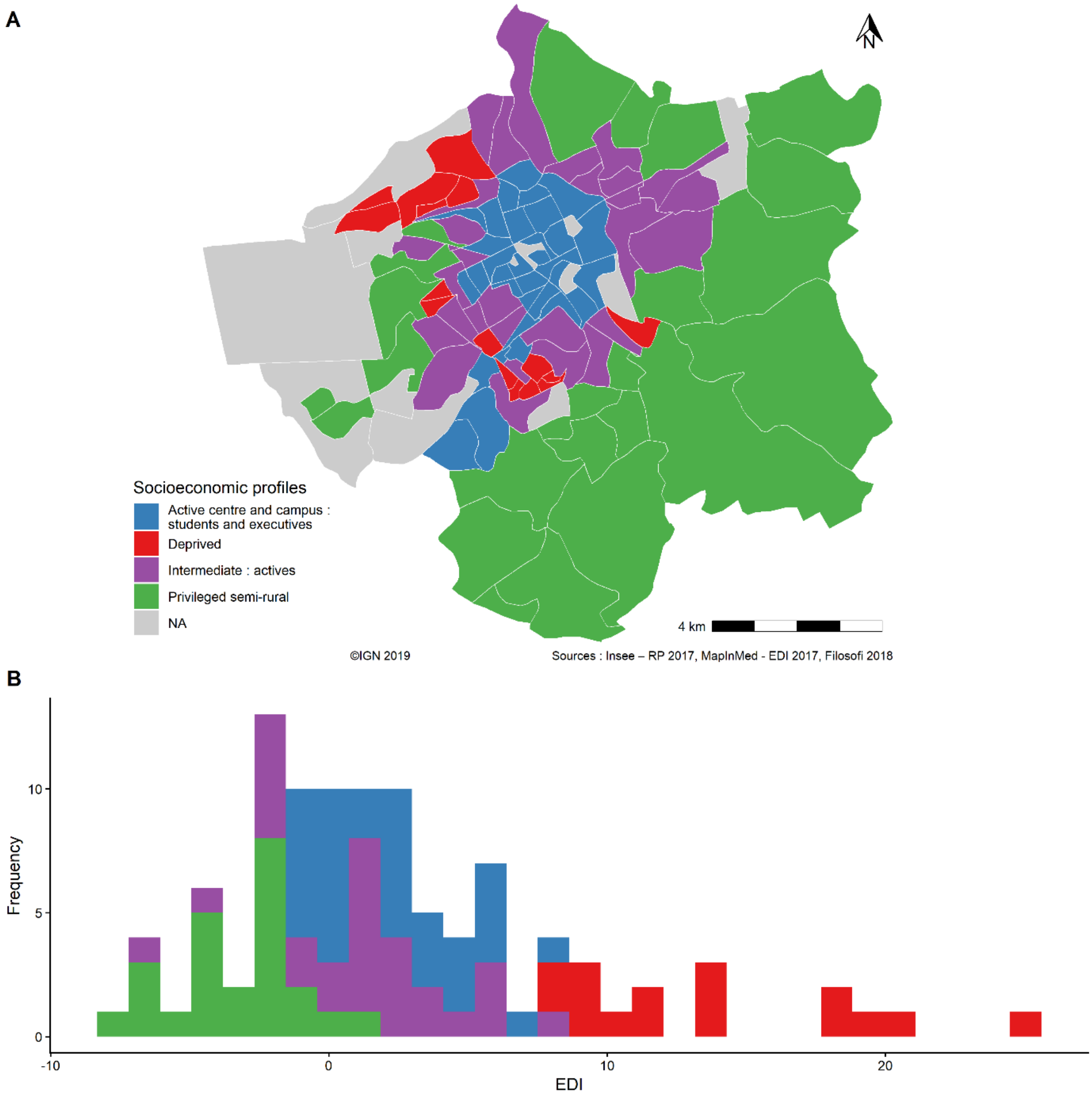

Fig 23: (A) Spatial distribution of the 4 socioeconomic profiles of Grand Nancy and (B) histogram of the European Deprivation Index (EDI), with the histogram coloured according to the profiles. privileged semi-rural (n=24), intermediate: actives (n=29), active centre and campus: students and executives (n=33), deprived (n=16)

Adjustment variables

Age distribution indicator

To create an indicator of the distribution of age, we selected 10 variables from the population census showing the percentage of inhabitants by age class (0–2,3-5,6-10,11-17,18-24,25-39,40-54,55-64,65-79,> 79). We calculed a Pearson assymetry coefficient with this formula [2] :

$$\frac{3 * (mean\ age - median\ age)}{standard\ deviation\ age}$$

We estimated the mean, median and standard deviation by taking the center of the ten age classes. The Pearson asymmetry coefficient for age was interpreted as follows : a coefficient closer to 0 indicated a more symmetric age distribution. A positive coefficient suggested that the IRIS population tended toward younger ages, while a negative coefficient showed that the IRIS population tended toward older ages.

Population density

The population density was estimated by using the map of IRIS and a grid map that gave us residential areas where households lived. We calculated the area for each IRIS covered by the tiles, then we divided the total population of IRIS by this area and their base map of grid data to estimate the population density by IRIS.

Access to healthcare variables

Variables on access to healthcare come from the database equipment of INSEE and the Research, Studies, Evaluation and Statistics Directorate (DREES). We considered variables for the accessibility of testing. For each IRIS, we calculated several distances to the nearest medical laboratory and pharmacy where a COVID-19 test could be performed. We also estimated the number of pharmacies and medical laboratories per IRIS. We had other indicators describing the access to healthcare in general as the number of primary healthcare per IRIS. We used the localised potential accessibility (LPA), which is estimated at the municipal level and for each person, the number of consultations per year, taking into account the distance and the time to travel to the nearest medical doctor. Finally, we had the number of retirement homes per IRIS.

First, we chose the LPA because, unlike the other variables, it takes into account the time travel to consult a medical doctor. Secondly, we kept the number of retirement homes because the COVID-19 indicators included the results of these places. To have another variable in the IRIS scale on the accessibility of tests, we calculated the Spearman correlation and looked at the indicators that were least correlated with the LPA, the number of retirement homes and the deprivation score (EDI). Using this method, we selected the number of pharmacies and medical laboratories per IRIS.

Environment profiles

The raster data of land cover available on Copernicus contained 44 variables (31). Firstly, we grouped variables by following the Corine Land Cover nomenclature (level 2) [3]. Secondly, we made an additional grouping of variables to distinguish the artificial surfaces, the urban fabric and the other artificial surfaces as industrial, commercial, transport units, mine dumps and construction sites. We also grouped agriculture areas, forests, semi-natural areas and wetlands. Finally, we deleted water bodies because of IRIS border rivers and could bias analyses. To summarise, for each IRIS, we constructed 3 variables indicating the percentage of the urban fabric, other artificial surfaces (industrial, commercial…) and non-artificial surfaces covering the IRIS. We built environment profiles with the same method as socioeconomic profiles. For each metropolitan area, we injected the 3 environmental variables into a PCA, then we performed an HAC on axes with at least 99% inertia.

Univariate analysis

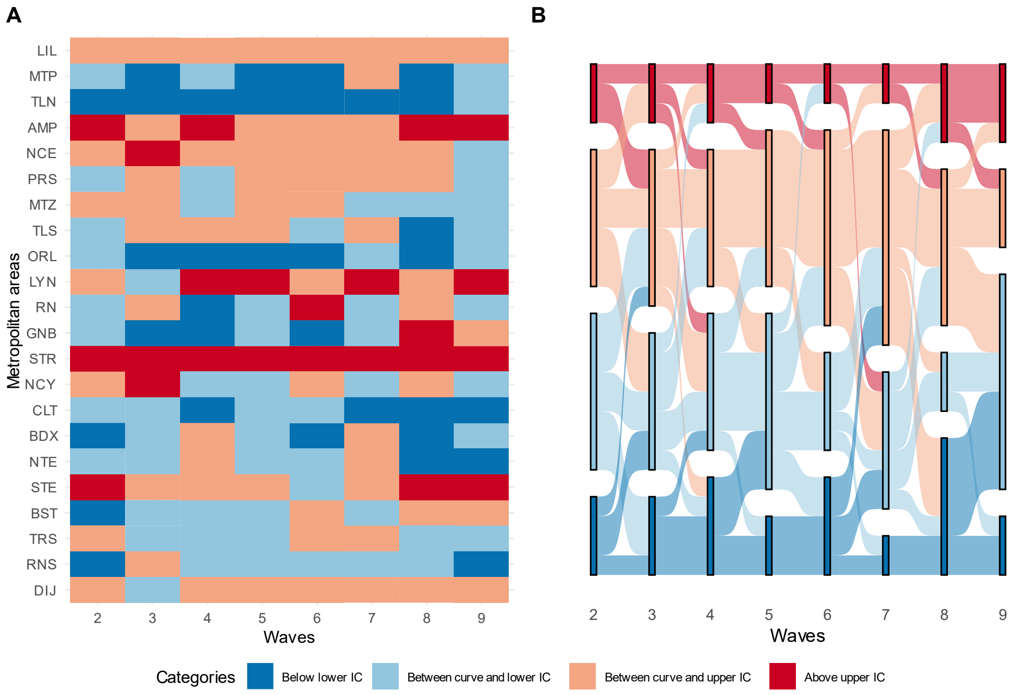

Fig 23: Heatmap (A) and Sankey diagram (B) showing the evolution by metropolitan area and wave of the categories of testing rates, as a function of the median EDI of the most deprived profiles in the metropolitan areas. The categories were defined based on their position relative to the adjustment curve derived from the LOESS regression and their confidence intervals (95% CI). On the heatmap (A), the metropolitan areas were ranked on the y-axis in ascending order of median EDI (Dijon had the lowest median EDI and Lille the highest). AMP = Aix-Marseille-Provence, BDX = Bordeaux, BST = Brest, CLT = Clermont Auvergne, DIJ = Dijon, GNB = Grenobles-Alpes, LIL = Lille, LYN = Lyon, MTP : Montpellier Méditerranée, MTZ = Metz, NCE = Nice Côte d'Azur, NCY = Grand Nancy, NTE = Nantes, ORL = Orléans, PRS = Grand Paris, RN = Rouen, RNS = Rennes, STE = Saint-Etienne, STR = Strasbourg, TLN = Toulon, TRS = Tours

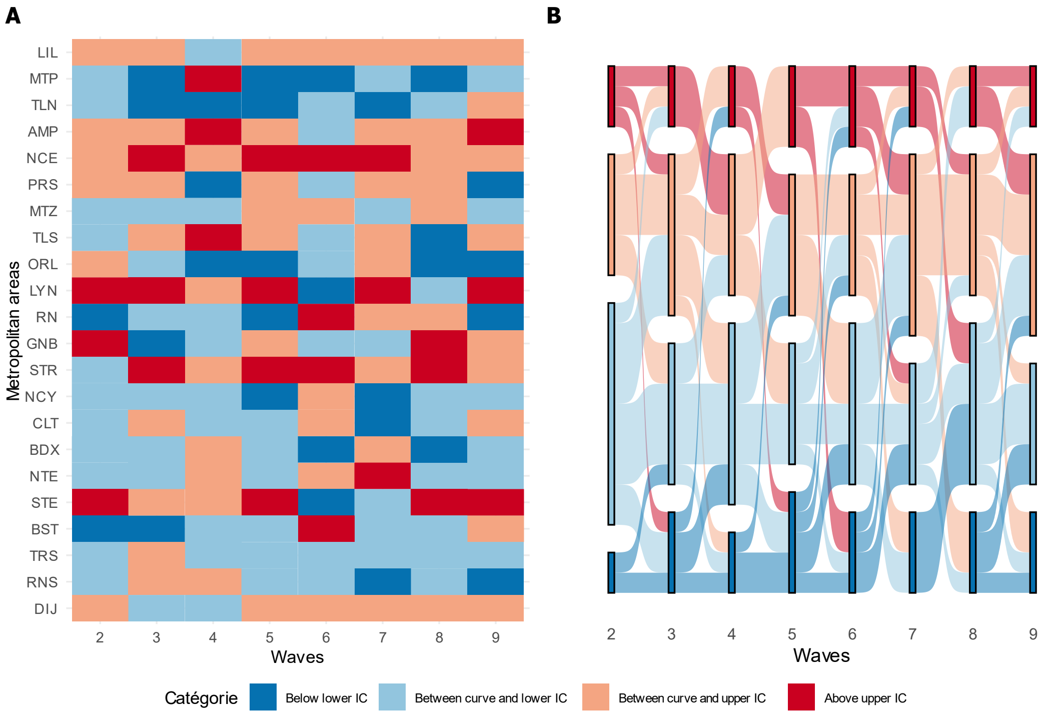

Fig 24: Heatmap (A) and Sankey diagram (B) showing the evolution by metropolitan area and wave of the categories of incidence rates, as a function of the median EDI of the most deprived profiles in the 22 French metropolitan areas. The categories were defined based on their position relative to the adjustment curve derived from the LOESS regression and their confidence intervals (95% CI). On the heatmap, the metropolitan areas were ranked on the y-axis in ascending order of median EDI (Dijon had the lowest median EDI and Lille the highest). AMP = Aix-Marseille-Provence, BDX = Bordeaux, BST = Brest, CLT = Clermont Auvergne, DIJ = Dijon, GNB = Grenobles-Alpes, LIL = Lille, LYN = Lyon, MTP : Montpellier Méditerranée, MTZ = Metz, NCE = Nice Côte d'Azur, NCY = Grand Nancy, NTE = Nantes, ORL = Orléans, PRS = Grand Paris, RN = Rouen, RNS = Rennes, STE = Saint-Etienne, STR = Strasbourg, TLN = Toulon, TRS = Tours

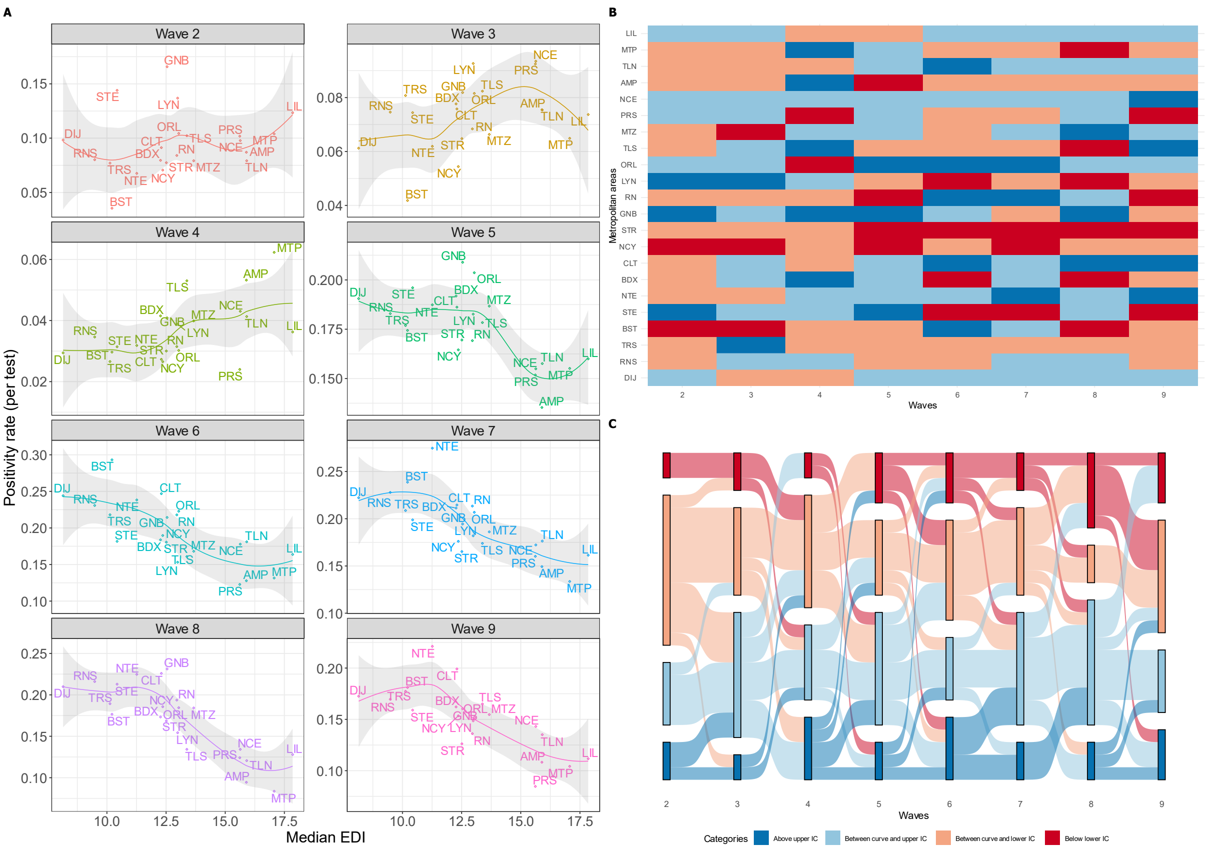

Fig 25: Evolution by wave (waves 2 to 9) of positivity rates compared with the median deprivation score for the most deprived IRIS in the 22 French metropolitan areas (A), France, July 2020 – March 2023, n=1096. The graphs illustrate the smoothing curves (LOESS regressions with 95% confidence intervals colored in grey). Heatmap (B) and Sankey diagram (C) showing the evolution by metropolitan area and wave of the categories of incidence rates, as a function of the median EDI of the most deprived profiles in the metropolitan areas. The categories were defined based on their position relative to the adjustment curve derived from the LOESS regression and their confidence intervals (95% CI). On the heatmap, the metropolitan areas were ranked on the y-axis in ascending order of median EDI (Dijon had the lowest median EDI and Lille the highest). AMP = Aix-Marseille-Provence, BDX = Bordeaux, BST = Brest, CLT = Clermont Auvergne, DIJ = Dijon, GNB = Grenobles-Alpes, LIL = Lille, LYN = Lyon, MTP : Montpellier Méditerranée, MTZ = Metz, NCE = Nice Côte d'Azur, NCY = Grand Nancy, NTE = Nantes, ORL = Orléans, PRS = Grand Paris, RN = Rouen, RNS = Rennes, STE = Saint-Etienne, STR = Strasbourg, TLN = Toulon, TRS = Tours

Models

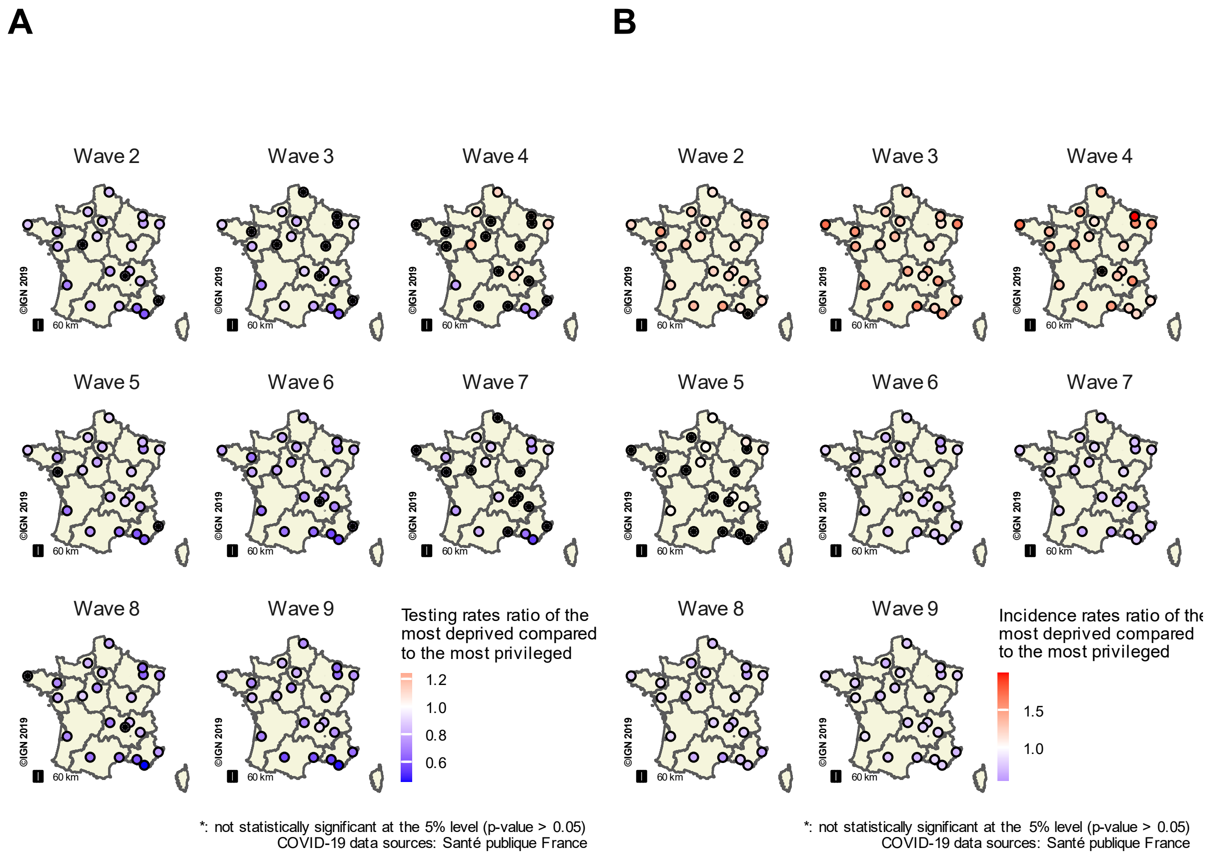

Fig 26: Spatial distribution of testing (A) and incidence (B) rate ratios between the most deprived and most privileged profiles in the 22 french metropolitan areas, by wave (waves 2 to 9).
The ratios are calculated from univariate (including random effects and spatial autocorrelation) GAM models per wave comparing testing and incidence rates for each metropolitan area between the socioeconomic profile with the lowest average EDI and the other profiles (French metropolitan areas, July 2020 – March 2023, n=7186). * : p-value > 0.05

Table 3: Testing rate ratios between the most deprived and most privileged profiles in the french metropolitan area of Nice, by wave (waves 2 to 9).

Waves	With IRIS outlier in Nice						Without IRIS outlier in Nice					
	Univariate (with spatial autocorrelation and random effects)			Multivariate			Univariate (with spatial autocorrelation random effects)			Multivariate		
	TRR	IC 95%	p-value	aTTR	IC 95%	p-value	TRR	IC 95%	p-value	aTRR	IC 95%	p-value
2	0.91	0.8 1.04	0.16	0.98	0.83 1.16	0.81	0.92	0.81 1.06	0.26	0.99	0.84 1.18	0.93
3	0.91	0.76 1.08	0.26	1.11	0.89 1.37	0.35	0.89	0.75 1.07	0.21	1.08	0.87 1.35	0.49
4	0.99	0.83 1.18	0.93	1.25	1 1.55	0.05	0.93	0.78 1.11	0.41	1.14	0.92 1.42	0.23
5	0.98	0.8 1.19	0.81	1.23	0.96 1.59	0.11	0.88	0.72 1.07	0.21	1.09	0.85 1.4	0.49
6	1.05	0.83 1.32	0.70	1.43	1.05 1.93	0.02	0.82	0.65 1.02	0.08	1.09	0.82 1.45	0.57
7	1.25	0.99 1.57	0.06	1.59	1.18 2.13	0	0.92	0.74 1.15	0.48	1.18	0.9 1.54	0.24

8	1.07	0.84 1.36	0.59	1.45	1.08 1.94	0	0.76	0.61 0.96	0.02	1.12	0.85 1.49	0.41
9	0.96	0.75 1.22	0.72	1.08	0.8 1.45	0.61	0.66	0.53 0.82	0	0.77	0.58 1.02	0.06

The ratios were calculated from univariate and multivariate GAM models per wave comparing testing rates for each metropolitan area between the socioeconomic profile with the lowest average EDI and the other profiles (Nice, July 2020 – March 2023, n=224).

Meta-regressions

A

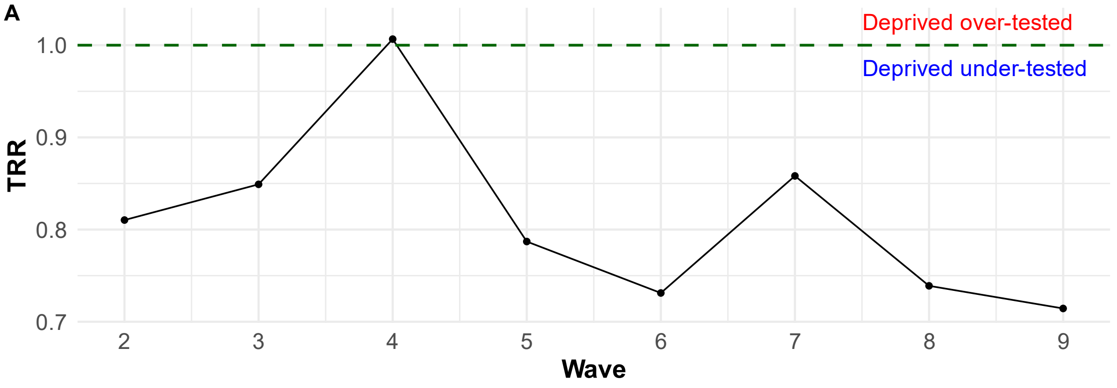

B

Variable	Beta	95% CI	p-value
Wave	-0.045	[-0.062, -0.029]	<0.001
Median cumulative vaccination rate (in %)	0.0020	[0.0008, 0.0033]	0.018
Median EDI of deprived profile	-0.020	[-0.029, -0.011]	<0.001

Fig 27: Mean of TRRs between the most deprived and most privileged profiles per epidemic wave (A) and results of meta-regression on logarithm of aTRRs in the 22 French metropolitan areas (B), July 2020 – March 2023 (n=176). Green dotted lines corresponded to the testing equality point between the privileged and the deprived (TRR = 1). The table showed estimated betas for the three explanatory variables (wave, median cumulative vaccination rate and median EDI of deprived profile) with confidence intervals (95% CI) and p-values.

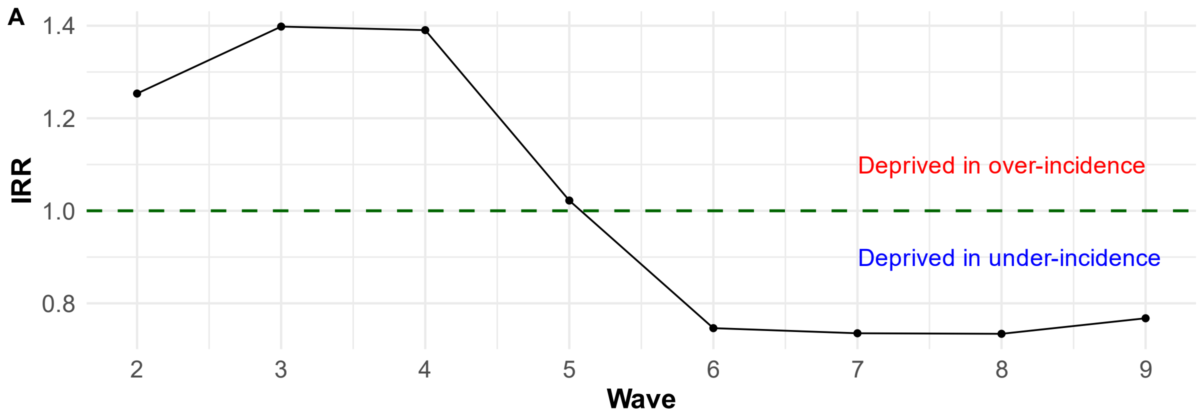

B

Variable	Beta	95% CI	p-value
Wave	-0.093	[-0.112, -0.074]	<0.001
Median cumulative vaccination rate (in %)	-0.0010	[-0.0025, 0.0004]	0.15
Median EDI of deprived IRIS	-0.008	[-0.018, 0.002]	0.11

Fig 29: Mean of IRRs between the most deprived and most privileged profiles per epidemic wave (A) and results of meta-regression on logarithm of IRRs in the 22 French metropolitan areas (B), July 2020 – March 2023 (n=176). Green dotted lines corresponded to green dotted lines corresponded to the testing equality point between the privileged and the deprived (IRR = 1). The table showed estimated betas for the three explanatory variables (wave, median cumulative vaccination rate and median EDI of deprived profile) with confidence intervals (95% CI) and p-values.

References :

1. Institut national de la statistique et des études économiques (INSEE). En quatre vagues, l'épidémie de Covid-19 a causé 116 000 décès et lourdement affecté le système de soins − France, portrait social | Insee [Internet]. 2021 [cited 2024 Feb 1]. Available from: https://www.insee.fr/fr/statistiques/5432509?sommaire=5435421#onglet-1

2. Weisstein EW. Pearson's Skewness Coefficients [Internet]. Wolfram Research, Inc.; [cited 2024 Sep 10]. Available from: https://mathworld.wolfram.com/

3. Accueil :: Cours Corine Land Cover [Internet]. [cited 2024 Feb 2]. Available from: https://land.copernicus.eu/content/corine-land-cover-nomenclature-guidelines/html/
